# Fundamental Properties of Low-Dimensional Perovskite-Related Light Absorbers: [(CH_2_)_3_NH_2_]_3_Sb_2_X_9_ (X = I, Br, Cl), Azetidinium Antimony Halides

**DOI:** 10.1021/acsomega.5c08306

**Published:** 2025-12-24

**Authors:** Young Un Jin, Bernd Marler, Andrei N. Salak, Marianela Escobar-Castillo, Lars Leander Schaberg, Erik Elkaïm, Niels Benson, Doru C. Lupascu

**Affiliations:** † Institute for Materials Science and Center for Nanointegration Duisburg-Essen (CENIDE), 27170 University of Duisburg-Essen, 45141 Essen, Germany; ‡ Institute of Geology, Mineralogy and Geophysics, 9142 Ruhr-University Bochum, 44780 Bochum, Germany; § Department of Materials and Ceramics Engineering, CICECO-Aveiro Institute of Materials, 56062 University of Aveiro, 3810-193 Aveiro, Portugal; ∥ Institute of Technology for Nanostructures (NST), University of Duisburg-Essen, 47057 Duisburg, Germany; ⊥ 55536 Synchrotron Soleil, L’Orme des Merisiers, Saint-Aubin, BP 48, 91192 Gif-sur-Yvette Cedex, France

## Abstract

Over the past decade, many Sb-based organohalides have been synthesized and analyzed due to their intriguing optical and electronic properties. They form low-dimensional (0D, 1D, and 2D) metal halide frameworks due to the 3+ oxidation state of antimony (Sb^3+^). The low-dimensional perovskite derivatives commonly have good moisture stability which makes them attractive for replacing the lead-containing halide perovskites. We focus on the A_3_Sb_2_X_9_ compositions since the band gap tunability of the compounds is noteworthy. Cs_3_Sb_2_I_9_ is known to form both 0D and 2D octahedral networks at room temperature. The A-site in A_3_Sb_2_X_9_ is crucial in determining the configuration of the metal halide framework. Within the hybrid A_3_Sb_2_X_9_ systems, the azetidinium cation (Az^+^, [(CH_2_)_3_NH_2_]^+^) has rarely been studied as a potential A-site candidate. We confirm that the polycrystalline powders and thin films of (Az)_3_Sb_2_I_9_ exhibit a 0D dimer structure, while (Az)_3_Sb_2_Br_9_ and (Az)_3_Sb_2_Cl_9_ adopt a 2D corrugated layer structure at the molecular level. (Az)_3_Sb_2_I_9_ and (Az)_3_Sb_2_Cl_9_ exhibit phase transitions at low temperatures, as shown by differential scanning calorimetry. Thin films grow with a strongly preferred (00*l*) orientation perpendicular to glass substrates. The Tauc plot analysis from diffuse reflectance spectra exhibits a band edge at 2.11 eV in (Az)_3_Sb_2_I_9_, at 2.63 eV in (Az)_3_Sb_2_Br_9_, and at 3.26 eV in (Az)_3_Sb_2_Cl_9_. A weak excitonic band is identified in the absorption spectrum of the (Az)_3_Sb_2_I_9_ thin film. Interestingly, there is a severe morphological difference in the thin films, depending on the solvent used, resulting in a band edge shift. The temperature dependence of the (Az)_3_Sb_2_I_9_ thin-film XRD data indicates the existence of an intermediate phase.

## Graphical abstract

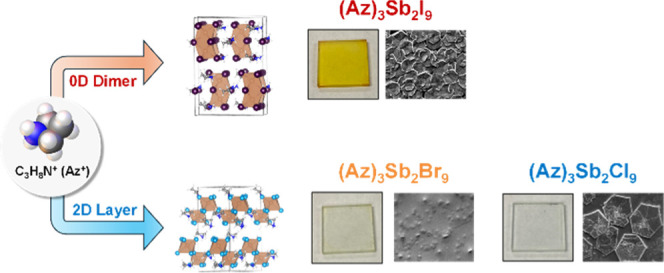

## Introduction

Hybrid halides have been dominating research in optoelectronics and photovoltaics in the past decade. The best known class is the perovskites of stoichiometry ABX_3_ (A = Cs, MA, or FA, B = Pb, Sn, X = I, Br, or Cl). The material design and development of hybrid lead halides APbX_3_ have driven a dramatic rise in power conversion efficiency (PCE) in photovoltaics.^1–4^ Research has proven that APbX_3_ possesses a large absorption coefficient, sharp optical absorption edge, low exciton binding energy, long electron–hole diffusion length, and tunability of its optical band gap.^5–9^ However, using APbX_3_ raises two big issues: (1) the material includes a toxic element Pb, and (2) it rapidly decomposes in air under light exposure and heat.^10–13^ These facts have led to the research of Pb-free hybrid perovskites. Many research groups have focused on ASnX_3_ and AGeX_3_.^14–17^ These materials still face a stability issue due to the oxidation of Sn and Ge from 2+ to 4+.^3,18,19^ In addition, the geometric distortion in Ge­(II) iodide octahedra, where iodide is the largest anion in the halide series, further contributes to their instability.^20^ Accordingly, the use of bismuth ions (Bi^3+^) or antimony ions (Sb^3+^) on the B-site of ABX_3_ has attracted attention.^21–25^ Bi^3+^ and Sb^3+^ have an isoelectronic configuration (*s^2^p^0^*) similar to Pb^2+^. They are thus considered to lead to optoelectronic properties as APbX_3_. However, the Bi^3+^- or Sb^3+^-based analogues of Pb-containing halides adopt A_3_B_2_X_9_ compositions because of the necessary charge neutrality.^21,22,25^ This results in a lower dimensionality of the [BX_6_]^3–^ octahedra configuration at the molecular level, which typically adopts 0D dimer or 2D corrugated layer perovskite derivatives. 0D dimers of A_3_B_2_X_9_ have an isolated [B_2_X_9_]^3–^ face sharing a face of each octahedron. In contrast, 2D corrugated layers have a low-density layered structure of [BX_6_]^3–^ sharing one interconnecting atom at the corner of each octahedron. Generally, A_3_B_2_X_9_ exhibits better stability than ABX_3_ in both 0D dimers and 2D corrugated layers due to its structural rigidity, thereby resisting phase transitions and chemical attack.^21,25^

**Table 1:** Comprehensive Comparison Presenting (Az)_3_Sb_2_X_9_ Properties

		(Az)_3_Sb_2_I_9_ (0D)		(Az)_3_Sb_2_Br_9_ (2D)		(Az)_3_Sb_2_Cl_9_ (2D)	
optical properties	band gap (powder)	Indirect – 2.11 eV		Indirect – 2.63 eV		Indirect – 3.26 eV	
		Direct – 2.26 eV		Direct – 2.82 eV		Direct – 3.50 eV	
	absorption onset of thin film	DMF – 2.42 eV		DMF – 2.96 eV		DMF – 3.49 eV	
		DMSO – 2.45 eV		DMSO – 2.92 eV		DMSO – 3.44 eV	
		GBL – 2.34 eV		GBL – 2.96 eV		GBL – 3.44 eV	
thermal properties	phase transition temperature point in DSC (powder)	cooling	heating	cooling	heating	cooling	heating
		6 °C	18 °C	-	-	–55 °C	–51 °C
	decomposition temperature point (powder)	230 °C		210 °C		220 °C	
	decomposition temperature point (thin film)	140 °C		130 °C		100 °C	

In the past few years, a variety of A_3_Bi_2_X_9_ and A_3_Sb_2_X_9_ compounds have been reported as potential semiconducting materials. The all-inorganic compounds K_3_Bi_2_I_9_ and Rb_3_Bi_2_I_9_ adopt a 2D corrugated layer structure, while Cs_3_Bi_2_I_9_ adopts a 0D dimer structure.^26–28^ On the other hand, K_3_Sb_2_I_9_, Rb_3_Sb_2_I_9_, and Cs_3_Sb_2_I_9_ also adopt a 2D corrugated layer structure.^29^ It has been revealed that Cs_3_Sb_2_I_9_ can form a 0D dimer polymorph depending on the synthesis conditions.^30^ In the case of hybrid halides, both (MA)_3_Bi_2_I_9_ and (MA)_3_Sb_2_I_9_ adopt 0D but (MA)_3_Sb_2_I_9_ can also have 2D corrugated layer polymorphism (MA = methylammonium, CH_3_NH_3_^+^).^21,31,32^ Both (NH_4_)_3_Bi_2_I_9_ and (NH_4_)_3_Sb_2_I_9_ adopt a 2D corrugated layer structure, the effective radius of NH_4_^+^ is smaller than that of K^+^.^24,26^ Accordingly, the effective ionic radius of a cation on the A-site of A_3_B_2_X_9_ becomes a critical factor in determining the dimensionality of a material.^33^ The anionic radius of the halides on the X-site is also decisive due to the large radius difference among I^–^, Br^–^, and Cl^–^. Cs_3_Bi_2_Br_9_ and Cs_3_Sb_2_Br_9_ adopt a 2D corrugated layer structure, and Cs_3_Bi_2_Cl_9_ adopts a 1D chain-like structure.^34–37^

A_3_Sb_2_X_9_ compounds face challenges such as lower conductivity and higher band gap; nevertheless, their nontoxic nature and promising stability make them an attractive and sustainable choice compared to Pb-containing halide materials. In photovoltaics, A_3_Sb_2_X_9_ has shown great potential for device fabrication as a light absorber.^29–31^ The 0D dimer (MA)_3_Sb_2_I_9_-based device achieved a PCE of 2.04% with the planar architecture solar cells produced by the single-step method by the solution process.^38^ On the other hand, the 2D corrugated layer (MA)_3_Sb_2_I_9–*x*_Cl_*x*_-based device shows a PCE of 3.34% with greater air stability than that of the 0D dimer (MA)_3_Sb_2_I_9_-based device.^39^ (NH_4_)_3_Sb_2_I_9_ also has large potential. Zuo and Ding have shown the good quality of the (NH_4_)_3_Sb_2_I_9_ film morphology and band gap tunability of (NH_4_)_3_Sb_2_I_9_ with halide engineering.^40^ Lamminen et al. reported a record of the best PCE, 6.4%, designing a mixed A-site cation and X-site halide system of the A_3_Sb_2_X_9_ (Cs_2.4_MA_0.5_FA_0.1_Sb_2_I_8.5_Cl_0.5_, FA = formamidinium, HC­(NH_2_)_2_^+^).^41^ However, achieving a high PCE with these compounds is still a big challenge. Although low-dimensional systems typically exhibit lower conductivity and reduced PCE due to poorer octahedral connectivity compared to ideal 3D perovskites, the finding of the low-dimensional system with a low band gap is important as they generally offer improved stability.^21,31,39^ Recently, A_3_Sb_2_X_9_ has also been studied for other optoelectronic applications, such as light-emitting devices (LEDs). Cs_3_Sb_2_X_9_ synthesized as a form of nanocrystals and microplatelets, exhibiting a decent light emission.^42–44^ For LED fabrication, the feasibility of Cs_3_Sb_2_X_9_ quantum dot layers/thin films with high photoluminescence quantum yield (PLQY) has been investigated.^45,46^ In addition, some groups reported the ferroelectricity of A_3_Sb_2_X_9_. (TMA)_3_Sb_2_Cl_9_ and (FA)_3_Sb_2_I_9_ exhibit ferroelectric properties in the low-temperature phases [TMA = trimethylammonium, (CH_3_)_3_NH^+^].^47–49^ Ji et al. reported a new class of A_3_Sb_2_X_9_, (NMPyr)_3_Sb_2_Cl_9–9*x*_Br_9*x*_ (NMPyr = *N*-methylpyrrolidinium) presenting outstanding ferroelectricity (5.2–7.6 μC/cm^2^).^50^

We introduce three lesser-known hybrid antimony halides in which the azetidinium molecular cation (Az^+^, [(CH_2_)_3_NH_2_]^+^) is located on the A-site of A_3_Sb_2_X_9_: (Az)_3_Sb_2_I_9_, (Az)_3_Sb_2_Br_9_, and (Az)_3_Sb_2_Cl_9_. Azetidinium is a heterocyclic molecule that contains three carbons and one nitrogen, consisting of a ring. Its effective cationic radius is roughly 250 pm, which is similar to that of MA^+^ and FA^+^ (Figure 1).^51^ It is revealed that Az^+^ features a ring-puckering motion at a specific temperature.^52^ In previous reports, we demonstrated that synthesizing (Az)_2_AgBiBr_6_ and (Az)_3_Bi_2_X_9_ is feasible.^53–55^ The orientation of Az^+^ in the crystal lattice is highly random in these materials. As an initial study, Luo et al. successfully synthesized (Az)_3_Sb_2_Cl_9_ and (Az)_3_Sb_2_Br_9_, reporting their ferroelastic properties.^56^ Recently, Liu et al. further investigated the synthesis feasibility of the (Az)_3_Sb_2_X_9_ crystals and conducted a systematic study of these materials.^57^ Pering et al. first reported (Az)­PbI_3_ applied in photovoltaic devices.^58^ They demonstrated great stability of (Az)­PbI_3_-based devices compared to Cs/MA/FA-based lead perovskites. However, overcoming the high band gap and low dimensionality is a significant challenge for photovoltaic applications.^58,59^ Rok et al. reported specific Az^+^ molecular dynamics inducing phase transitions at low temperatures in a new 1D (Az)­CdCl_3_ perovskite-like compound.^60^ We synthesized polycrystalline (Az)_3_Sb_2_X_9_ adopting a low-dimensional system at the molecular level of the [SbX_6_] octahedra network and successfully deposited their thin films on glass substrates. This yields the necessity of studying the correlation between the Az^+^ dynamics and structure properties.

**Figure 1:**
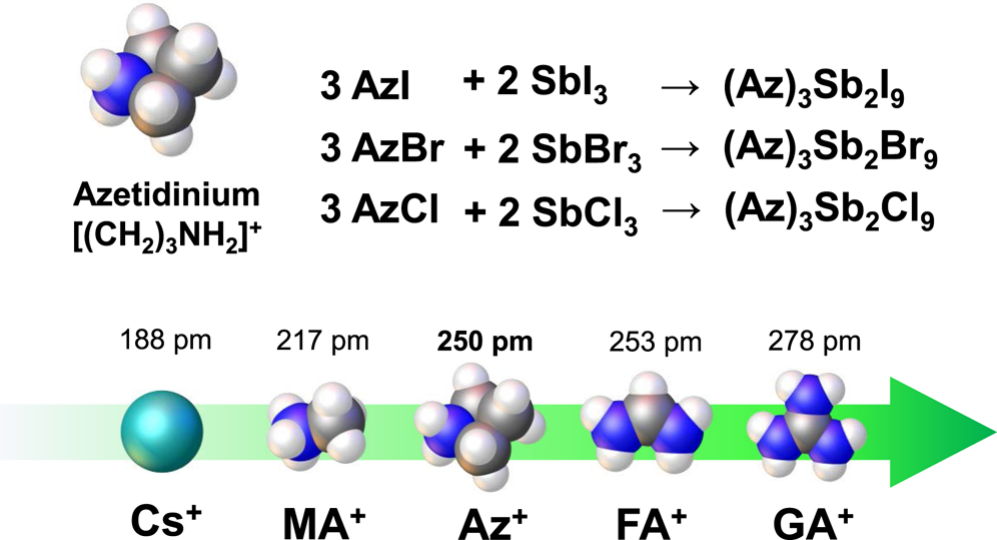
Depiction of the azetidinium cation (grey spheres: carbon atoms, blue sphere: nitrogen atoms, white spheres: hydrogen atoms), chemical reactions to form (Az)_3_Sb_2_X_9_, and the similarity of effective ionic radii of Cs^+^, MA^+^, FA^+^, and GA^+^ (MA = methylammonium, FA = formamidinium, and GA = guanidinium).

## Experimental Section

### Polycrystalline Powder Synthesis

Azetidine hydrochloride (AzCl, 97%) was purchased from Sigma-Aldrich. Azetidinium bromide (AzBr) and azetidinium iodide (AzI) were synthesized by the reaction of azetidine and hydrohalic acids (HBr of 48%, and HI of 55–58%).^54^ Azetidine (98%) was purchased from Thermo Scientific Chemicals. Antimony­(III) iodide (SbI_3_, 98%) was purchased from Sigma-Aldrich. Antimony­(III) bromide (SbBr_3_, 99%) was purchased from Alfa Aesar. Antimony­(III) chloride (SbCl_3_, ACS reagent) was purchased from ACROS Organics.

(Az)_3_Sb_2_X_9_ polycrystalline powders with high purity were synthesized by using an evaporation method (Figure 1). Every precursor was filtered by a PTFE membrane filter of 0.7 μm pore size. For the polycrystalline powder of (Az)_3_Sb_2_I_9_, we used acetonitrile to dissolve AzI and SbI_3_ and made 0.1 M of the precursor. A bright-orange colored powder was obtained by evaporation at 50 °C on a hot plate in a fume hood. We then recrystallized the powder in acetonitrile again. The polycrystalline powder of (Az)_3_Sb_2_Br_9_ was synthesized from 0.2 M precursor dissolved in dimethylformamide (DMF) by evaporation at 60 °C in a vacuum oven and recrystallized in absolute ethanol in a N_2_-filled glovebox. The (Az)_3_Sb_2_Cl_9_ polycrystalline powder was obtained from a 0.2 M precursor dissolved in DMF by evaporation at 40 °C in a vacuum oven and recrystallized in absolute ethanol in a fume hood. In the case of the chloride, the dissolution in ethanol was complete, for the bromide it was only partial. Every synthesized crystal appeared unevenly grown, twinned, nonuniform, or in a disordered state.

### Thin-Film Deposition

Thin-film deposition was performed by spin-coating in a N_2_-filled glovebox. Normal glass substrates of 1.5 cm × 1.5 cm were used. The substrates were cleaned with acetone, distilled water, and ethanol. After that, an UV–ozone treatment was performed for 30 min. Spin-coating parameters were 2000 rpm for 30 s. 0.3 M precursors for (Az)_3_Sb_2_X_9_ dissolved in DMF, dimethyl sulfoxide (DMSO), and γ-butyrolactone (GBL) were used. The precursors were filtered through a PTFE membrane filter of 0.7 μm pore size. The used reactants are identical to the ones used for the synthesis of the polycrystalline powders. The annealing temperature of (Az)_3_Sb_2_I_9_ and (Az)_3_Sb_2_Br_9_ was 100 °C for 30 min on a hot plate in a N_2_-filled glovebox, while the one of (Az)_3_Sb_2_Cl_9_ was 75 °C for 30 min on a hot plate in a N_2_-filled glovebox.

### Synchrotron XRD Measurements

Synchrotron XRD measurements of the polycrystalline powders were performed on the CRISTAL beamline at the SOLEIL synchrotron facility. The powder diffractograms were recorded using a two-circle diffractometer at a beam energy of ca. 21 keV corresponding to a wavelength of 0.58244 Å. A curved pixel detector, 9x Dectris Mythen II modules, positioned on a cylinder with a radius of 720 mm, and a Si(111)-double crystal monochromator were used. The data sets were collected in the transmission mode with small quantities of the samples enclosed in a horizontally oriented rotating glass capillary of 0.5 mm thickness.

### Thin-Film XRD Measurement

The laboratory XRD study of the thin films was carried out using a PANalytical X’pert PRO diffractometer (Cu Kα_1/2_ radiation) equipped with a PIXcel3D-Medipix3 detector. Pole figures were obtained using a Euler chi-phi xyz stage of 240 mm for each reflection peak of thin films fixed onto the center of the stage. The continuous scanning mode was used for every orientation, of which the polar angle χ (chi) ranged from 0° to 90° with 1° steps, and the azimuthal angle φ (phi) was scanned over 0° to 360° for 1 s per 1° step. Temperature-dependent XRD data of the thin films were collected in the range of 20–200 °C with a step of 10 °C using a Buehler HDK 2.4/SO stage with a Pt heat strip in ambient atmosphere. At each measuring point, the temperature was stabilized for 10 min before XRD data collection.

## Results and Discussion

### Crystal Structure

The crystal structure of (Az)_3_Sb_2_X_9_ was solved by direct methods and subsequently refined by the Rietveld method using the polycrystalline powder XRD data collected using high energy monochromatic synchrotron X-ray radiation.

According to our structure refinement, (Az)_3_Sb_2_I_9_ adopts an orthorhombic structure with space group 63 (*Cmcm*) (see Figure 2, and Table S1). The structure diagram shows a pseudohexagonal structure which is nearly identical to that of (Az)_3_Bi_2_Br_9_.^54^ Two [SbI_6_] octahedra share one common face, thereby forming a 0D dimer. The Az^+^ molecular coordinate was accommodated by allocating four carbon atoms in the free space. For the refinement of Az^+^, only carbon atoms were used instead of the correct composition, C_3_H_8_N^+^, because the molecular orientational disorder would imply several unnecessary refinement complications. The intercalated Az^+^ cations surrounding the [SbI_6_] dimers are randomly rotated in the crystal lattice.

**Figure 2:**
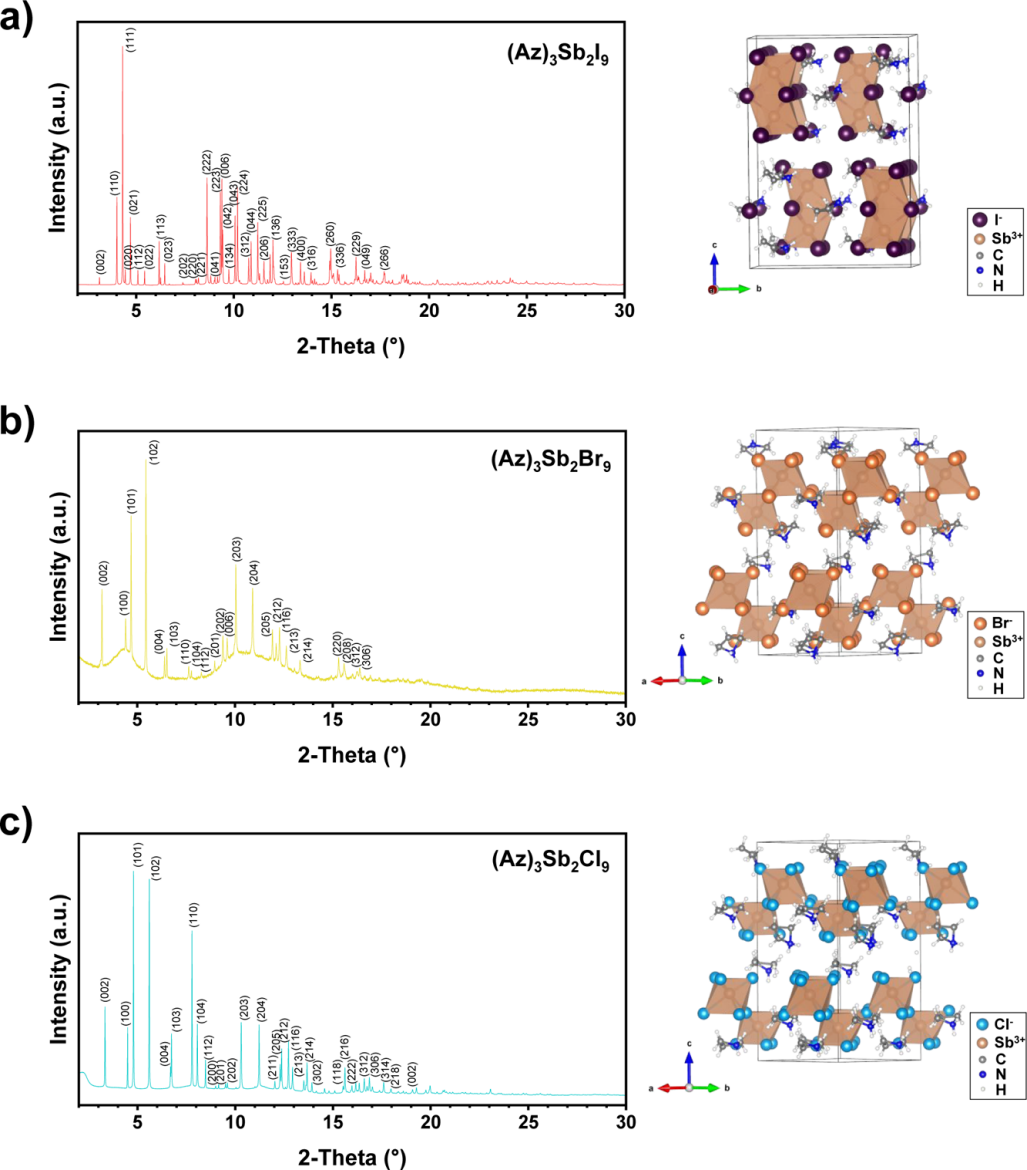
Refined synchrotron PXRD patterns and polyhedral representations of (a) (Az)_3_Sb_2_I_9_, (b) (Az)_3_Sb_2_Br_9_, and (c) (Az)_3_Sb_2_Cl_9_. The azetidinium cations are highly disordered in the cationic lattice. The polyhedral representation of (Az)_3_Sb_2_Cl_9_ does not contain the residual H_3_O^+^ ions used in refinement.

(Az)_3_Sb_2_Br_9_ adopts a trigonal structure with space group 159 (*P*31*c*), containing 2D corrugated layers. The polyhedral network of (Az)_3_Sb_2_Br_9_ appears similar to its analogues, Cs_3_Sb_2_Br_9_ and (Az)_3_Bi_2_Cl_9_.^34,54^ The material includes (semi)­amorphous phase(s), as indicated by the intense and broad background humps around 4.5° and 10.5° (Figure S1).

Liu et al. reported that (Az)_3_Sb_2_I_9_ adopts *Cmcm* space group,^57^ while Luo et al. indicate that (Az)_3_Sb_2_Br_9_ and (Az)_3_Sb_2_Cl_9_ adopt the *P*6_3_ *mc* space group.^56^

In contrast, we initially refined the structures in all cases based on a subgroup: *Cmc*2_1_ instead of *Cmcm* for (Az)_3_Sb_2_I_9_, and *P*31*c* instead of *P*6_3_ *mc* for (Az)_3_Sb_2_Br_9_ and (Az)_3_Bi_2_Cl_9._ These subgroups have fewer symmetry elements, leading to a larger positional freedom of certain atoms (e.g., Br and Cl) within the structure. In the given cases, the subgroups (*Cmc*2_1_ and *P*31*c*) have the same rules for systematically extincted reflections as the corresponding supergroups (*Cmcm* and *P*6_3_ *mc*), making it difficult to definitively distinguish between corresponding space groups using powder XRD data. Accordingly, to determine the most probable space group symmetry of the (Az)_3_Sb_2_X_9_ materials, we eventually refined the structures assuming both higher symmetry (*Cmcm* or *P*6_3_ *mc*) and lower symmetry (*Cmc*2_1_ or *P*31*c*) based on our synchrotron data. The refinements show that the structure-related *R* values, *R*(*I*), are significantly smaller for the lower-symmetry models of (Az)_3_Sb_2_Br_9_ and (Az)_3_Sb_2_Cl_9_ (see Table S2). Also, profile-related values of *R*(wp) and Chi^2^ pointed to the lower symmetry (Table S2). Even though it cannot be guaranteed that these two materials do not crystallize in *P*6_3_ *mc*, we decided—lacking superior single-crystal data but based on the improved *R* values—to present these structures with space group symmetry *P*31*c*. For (Az)_3_Sb_2_I_9_, both refinements yielded nearly identical *R*(*I*), *R*(wp), and Chi^2^ values, and since the *Cmcm* refinement required fewer variable parameters, it was considered preferable.

Comparing the room-temperature structures reported by Luo et al. with our structure refinements, we find the two structures of (Az)_3_Sb_2_Br_9_ to be nearly identical—in spite of slightly different symmetries.^56^ The [SbCl_6_] octahedra of (Az)_3_Sb_2_Cl_9_ refined in *P*6_3_ *mc*, however, are significantly more regular than those in our structure. As we applied the same soft distance restraints on Sb-X and X···X and the same symmetry for the two refinements (X = Br, Cl), the pronounced distortion observed in the [SbCl_6_] octahedra of our (Az)_3_Sb_2_Cl_9_ structure is, presumably, not symmetry related but probably related to the presence of some hydronium ions in our material.

The structure contains three independent Az^+^ sites (all of them are two-fold sites). Electron density difference maps determined during the course of the refinement showed that two of these sites are occupied by Az^+^ cations which assume a certain position but are rotationally disordered about a fixed center (see Figure S2). These are simulated in the refinement by six carbon atoms with a fixed occupancy factor that covers the complete scattering power (i.e., all electrons) of the Az^+^ cation with composition C_3_H_8_N^+^. Surprisingly, the electron density cloud representing the third Az^+^ site was abnormally elongated. The electron density could not be described by only Az^+^ cations. The most convincing result when refining the structure of the (Az)_3_Sb_2_Cl_9_ compound was obtained by assuming that a part of the Az^+^ cations at this particular site are replaced by the hydronium (H_3_O^+^) ion. The refinement of the occupancy factors indicates that this site is shared by Az^+^ and H_3_O^+^, leading to a composition of (C_3_H_8_N)_5.2_(H_3_O)_0.8_[Sb_4_Cl_18_] per unit cell (see Table S1). We assume that the H_3_O^+^ ions are incorporated during the recrystallization of (C_3_H_8_N)_6_[Sb_4_Cl_18_] in ethanol conducted to enhance the crystallinity of the material. This will be described in the thin-film section supporting that hypothesis. The refined (Az)_3_Sb_2_Cl_9_ adopts a trigonal structure with space group 159 (*P*31*c*), which also forms 2D corrugated layers. The structure of the compound is almost equal to that of (Az)_3_Sb_2_Br_9_.

### FT-IR Analysis

FT-IR transmittance spectra of polycrystalline powders were measured to check the presence of the Az^+^ ring in the crystal lattice (Figure S3). The spectra show characteristic frequencies of the azetidine ring, similar to our previously reported spectra of (Az)_3_Bi_2_X_9_.^54^ When Az^+^ is incorporated in the crystal system (Az)_3_Sb_2_X_9_, its interactions with the antimony-halogenide framework influence its vibrational modes. Peak shifting and changes in the peak intensity reflect this interaction. It can affect the hydrogen bonding (N–H stretching) and the local structural strain, altering the C–H and C–N stretching and bending modes. In the FT-IR spectra, the clear peak in the 625–683 cm^–1^ range is attributed to the azetidine ring deformation mode.^61,62^ The peaks in the range of 2964–3067 cm^–1^ can be assigned to the C–H stretching vibrations.^61,62^ The N–H stretching mode can be seen as a broad peak at around 3400 cm^–1^.^61,62^

### Thin-Film Properties

Thin-film deposition of (Az)_3_Sb_2_X_9_ was performed on cleaned glass substrates via spin-coating in a N_2_-filled glovebox. The glass substrates were cleaned using ultrasonication in acetone for 15 min, followed by distilled water for 15 min, and finally in ethanol for 15 min. We chose three different solvents to obtain the precursor solutions: DMF, DMSO, or GBL. Each thin film of (Az)_3_Sb_2_X_9_ is named (Az)_3_Sb_2_X_9_-solvent (DMF/DMSO/GBL) according to the solvent used.

#### (Az)_3_Sb_2_I_9_

The (Az)_3_Sb_2_I_9_-DMF and (Az)_3_Sb_2_I_9_-DMSO thin films show hexagonal grain morphology, optically appearing slightly conical on the surface and a nearly complete surface coverage. The (Az)_3_Sb_2_I_9_-GBL thin film exhibits incomplete coverage and less structured flakes resembling carnation flowers (Figure 3a). (Az)_3_Sb_2_I_9_-DMSO develops larger grain sizes than (Az)_3_Sb_2_I_9_-DMF. The crystals of (Az)_3_Sb_2_I_9_-GBL appear to have irregular growth and a more obvious island formation. The XRD patterns commonly exhibit a dominant (006) reflection with a (00*l*) preferred orientation (Figure 3b). Different solvents generate distinctly dissimilar crystallinity of the films (Figure S4a). The most intense reflections are detected in (Az)_3_Sb_2_I_9_-DMSO due to its large grain size and good surface coverage. The peak intensity of (Az)_3_Sb_2_I_9_-GBL at (006) is higher than that of (Az)_3_Sb_2_I_9_-DMF. This is caused by the larger crystal size of (Az)_3_Sb_2_I_9_-GBL than that of (Az)_3_Sb_2_I_9_-DMF. The pole figure of the dominant (006) peak of (Az)_3_Sb_2_I_9_-DMSO is investigated because its morphology and crystallinity are the best (Figure 3c).

**Figure 3:**
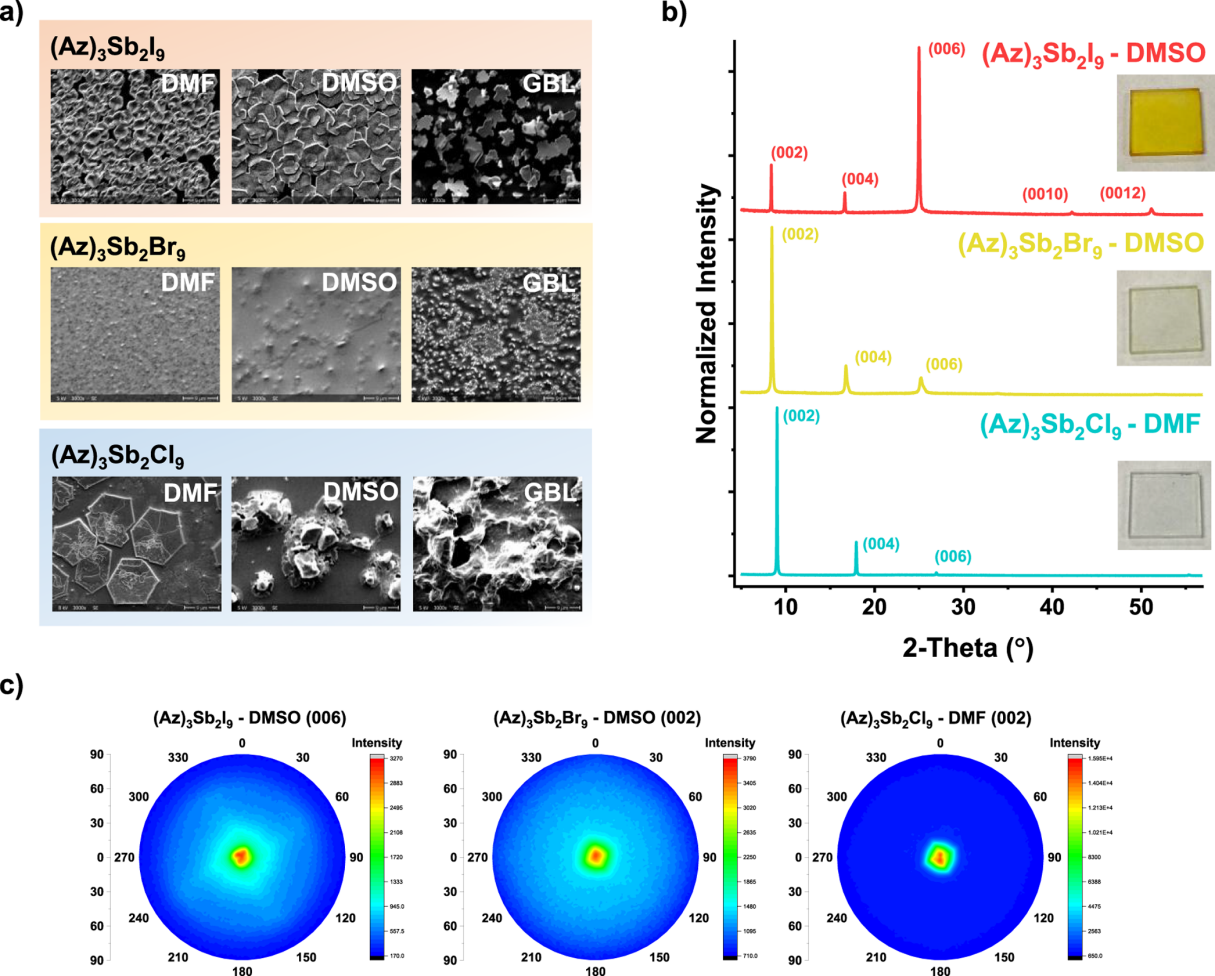
(a) SEM pictures of (Az)_3_Sb_2_X_9_ thin films on a cleaned glass substrate depending on the solvent used in the precursors (DMF, DMSO, and GBL), (b) XRD patterns and photo of (Az)_3_Sb_2_I_9_-DMSO, (Az)_3_Sb_2_Br_9_-DMSO, and (Az)_3_Sb_2_Cl_9_-DMF thin films on glass. (c) Pole figures of the (006) reflection of (Az)_3_Sb_2_I_9_-DMSO, (002) reflection of (Az)_3_Sb_2_Br_9_-DMSO, and (002) reflection of (Az)_3_Sb_2_Cl_9_-DMF.

#### (Az)_3_Sb_2_Br_9_

(Az)_3_Sb_2_Br_9_ thin films are typically tinged with a yellowish transparent color. The SEM pictures show that the morphological difference is obvious depending on which solvent is used for the precursors (Figure 3a). (Az)_3_Sb_2_Br_9_-DMF seems to have a grain distribution on its uneven surface; however, grain boundaries are not well visible. The XRD pattern shows almost no reflections, indicating a highly amorphous state (Figure S4b). (Az)_3_Sb_2_Br_9_-DMSO yields a denser layer formation with the growth of tiny, granulated crystals underneath. The XRD pattern shows an intense (002) reflection followed in intensity by the (004) and (006) reflections (Figure 3b). (Az)_3_Sb_2_Br_9_-GBL contains predominantly granulated crystals. It seems that the film partially consists of planar like crystals. The pole figure yields a strong (002) reflection for (Az)_3_Sb_2_Br_9_-DMSO, which also shows a highly textured (00*l*) out-of-plane orientation.

#### (Az)_3_Sb_2_Cl_9_

All of the (Az)_3_Sb_2_Cl_9_ thin films show an incompletely covered layer. Crystallization of (Az)_3_Sb_2_Cl_9_ is more pronounced in (Az)_3_Sb_2_Cl_9_-DMF (Figure 3a). The crystal shape appears to be hexagonal in the SEM image. Moreover, seemingly inhomogeneous ultrathin layers are observed on the crystal surface. We assume that it is imperfect and is layered growth. The (002) reflection is dominant in the XRD pattern (Figure 3b). We observed that the material does not exhibit good solubility in DMSO and GBL. In the SEM morphologies of (Az)_3_Sb_2_Cl_9_-DMSO and (Az)_3_Sb_2_Cl_9_-GBL, it is difficult to see crystals like those of (Az)_3_Sb_2_Cl_9_-DMF. (Az)_3_Sb_2_Cl_9_-DMSO shows (002) and (004) reflections, although this has low crystallinity in its XRD pattern (Figure S4c). The reflections from (Az)_3_Sb_2_Cl_9_-GBL are extremely low, which indicates that most of the material is amorphous. The (002) reflection is detected at approximately 9.00° 2θ (Cu Kα_1/2_ radiation) for every (Az)_3_Sb_2_Cl_9_ film. In contrast, the synchrotron data used for the refinement of the (Az)_3_Sb_2_Cl_9_ powder yield a 2θ position of the (002) reflection at 8.85° if converted by using Cu Kα_1/2_ (Figure S5). As mentioned above, our (Az)_3_Sb_2_Cl_9_ polycrystalline powder is best fitted with a unit cell corresponding to the composition (C_3_H_8_N)_5.2_(H_3_O)_0.8_[Sb_4_Cl_18_]. The difference in lattice dimensions suggests that all thin films form a layered structure with a complete (Az)_3_Sb_2_Cl_9_ stoichiometry, without incorporation of H_3_O^+^. The pole figure of the (002) orientation shows that it is highly textured with a strongly preferred growth (Figure 3c).

### UV Irradiation Stability

The UV light stability of the (Az)_3_Sb_2_X_9_ thin films has been examined. Every sample was exposed to an UV wavelength of 365 nm with 20 W/cm^2^ for 18 h in a fume hood. We recorded the XRD patterns of (Az)_3_Sb_2_I_9_-DMSO, (Az)_3_Sb_2_Br_9_-DMSO, and (Az)_3_Sb_2_Cl_9_-DMF since they represent the highest crystallinity in each composition. The recording was performed after 5, 9, 13, and 18 h of exposure (Figure S6a–c). The change in the intensity of the main reflections of the XRD patterns is organized in one graph: (006) of (Az)_3_Sb_2_I_9_-DMSO, (002) of (Az)_3_Sb_2_Br_9_-DMSO, and (002) of (Az)_3_Sb_2_Cl_9_-DMF (Figure S6d–f). The (006) reflection of (Az)_3_Sb_2_I_9_-DMSO exhibits a radical intensity decrease after 5 h and a slightly lower 2θ value. The 2θ value stabilizes, and no longer shifts after 9 h. The intensity steadily decreases up to 18 h. It then nearly disappears. The material likely decomposes. It is ascertained that (Az)_3_Sb_2_Br_9_-DMSO is stable under UV-light exposure. In 5 h, the intensity of the (002) reflection of (Az)_3_Sb_2_Br_9_-DMSO shows a slight increase and a decrease after 9 h. It is remarkable that an enhancement of crystallinity occurs. There is no shift in 2θ identified. The (Az)_3_Sb_2_Cl_9_-DMF shows a constant decline in the reflection intensity with UV light exposure. At 10.2°, one unknown peak is detected after 5 h and increases with UV light exposure time. We did not observe any shift of peaks for any reflection in (Az)_3_Sb_2_Cl_9_-DMF.

### Thermal Properties

#### Thermogravimetry (TG) and Differential Scanning Calorimetry Curve

We measured the TG curves of all powder samples with a 10 °C/min heating rate up to 800 °C (Figure 4a–c). Since the measurement was performed in argon flow, the initial mass % is detected over the value of 100% due to a buoyancy effect of the apparatus. (Az)_3_Sb_2_I_9_ displays a strong mass loss of around 79% from 230 °C. (Az)_3_Sb_2_Br_9_ shows a strong mass loss from 210 °C on, while (Az)_3_Sb_2_Cl_9_ shows a first strong mass loss above 220 °C. The first rapid mass losses of the materials are caused by decomposition because the mass loss is basically over 60%. The subsequent mass loss is considered to result from combustion.

**Figure 4:**
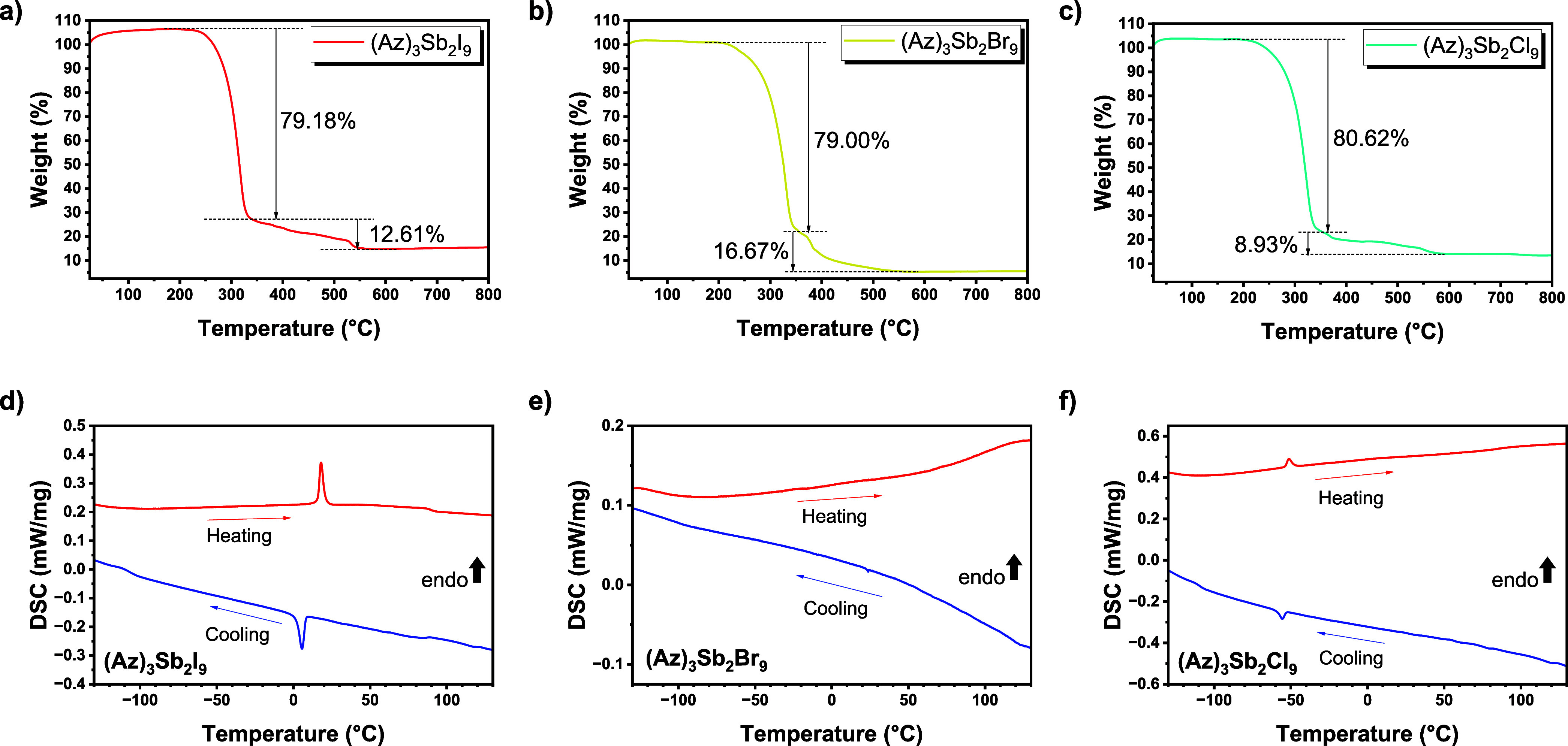
TG curve of the polycrystalline powder of (a) (Az)_3_Sb_2_I_9_, (b) (Az)_3_Sb_2_Br_9_, and (c) (Az)_3_Sb_2_Cl_9_ in the temperature range of decomposition. DSC curve of the polycrystalline powders of (d) (Az)_3_Sb_2_I_9_, (e) (Az)_3_Sb_2_Br_9_, and (f) (Az)_3_Sb_2_Cl_9_.

The DSC curves of (Az)_3_Sb_2_X_9_ were obtained in the temperature range from −130 to 130 °C, maintaining a rate of 10 °C/min (Figure 4d–f). First of all, we did not observe specific heat anomalies in (Az)_3_Sb_2_Br_9_ for both cooling and heating. The DSC curve of (Az)_3_Sb_2_I_9_ displays a phase transition temperature point at 6 °C in the cooling direction and 18 °C in the heating direction. The calculated Δ*H* at 6 °C in the cooling direction indicates −5.86 kJ/mol, and the Δ*S* is around −21.02 J/(mol K). The Δ*H* at 18 °C in the heating direction is calculated as 5.15 kJ/mol, and the Δ*S* is around 17.69 J/(mol K). Overall, we thus observe a first-order phase transition here. The DSC curve of (Az)_3_Sb_2_Cl_9_ shows a phase transition temperature point at −55 °C in the cooling direction and −51 °C in the heating direction. Δ*H* is calculated as −1.12 kJ/mol, which is smaller than the one of (Az)_3_Sb_2_I_9_ at −55 °C in the cooling direction. The Δ*S* is around −5.12 J/(mol K). At −51 °C in the heating direction, the Δ*H* is 0.75 kJ/mol and the Δ*S* is around 3.37 J/mol K.

We note that (Az)_3_Sb_2_Cl_9_ also has an obvious phase transition of first order but at a lower temperature than the iodide. The phase transition temperature points of the (Az)_3_Sb_2_I_9_ and (Az)_3_Sb_2_Cl_9_ samples are nearly identical to those reported previously.^56,57^ This further supports our assumption that H_3_O^+^ is present only in the recrystallized sample used for the structure analysis. Surprisingly, (Az)_3_Sb_2_Br_9_ does not show any phase transition point in the temperature range investigated, although its crystal symmetry is close to that of the chloride. Notably, previously reported (Az)_3_Sb_2_Br_9_ samples have shown a distinct phase transition temperature point.^56,57^ This difference can be attributed to the synthesis methods. Our synthesis involved a chemical reaction between antimony bromide and the azetidinium bromide salt in DMF, which had been synthesized in a previous step, whereas the other two groups used a conventional solution method where azetidine reacts in an acidic solution of antimony bromide.^56,57^ Our two-step synthesis may have affected crystallinity, resulting in a semiamorphous phase. We hypothesize that this semiamorphous state suppresses the cooperative atomic movements required for a phase transition, rendering it undetectable. (Az)_3_Sb_2_I_9_ appears to experience a higher symmetry change due to the larger observed change in entropy shown. The TG/DSC curves of (Az)_3_Sb_2_Cl_9_ show not even traces of impurities such as water or organic molecules other than azetidinium. These results support the idea that the H_3_O^+^ ion is only incorporated into the unit cell of the material used for structure analysis.

### Temperature-Dependent Thin-Film XRD

We also applied XRD to study the temperature behavior of the thin films. We selected one sample of each composition with high crystallinity and good substrate coverage, namely, (Az)_3_Sb_2_I_9_-DMSO, (Az)_3_Sb_2_Br_9_-DMSO, and (Az)_3_Sb_2_Cl_9_-DMF.

For the (Az)_3_Sb_2_I_9_-DMSO film, we focused on the (002) and (006) reflections. The shift of the (006) peak up to 24.00° corresponds to the increase of the respective interplanar distance from 3.5633(1) to 3.6933(8) Å (Figure 5a). The peak intensity decreases with increasing temperature up to 80 °C but increases between 80 and 120 °C. At 140 °C, the intensity of the (006) reflection strongly reduces, and the peak disappears at 180 °C. It was found that all other reflections vanish at 180 °C. Therefore, the decomposition practically begins between 130 and 140 °C. A slight peak splitting is seen at 60 °C, becoming one intense reflection at 110 °C. The intensity increases between 80 and 120 °C is thus estimated as an intermediate chemical composition formed by decomposition. The (002) reflection gradually increased in intensity up to 130 °C (Figure 5b). The d-value shift of (002) is approximately 0.49 Å which is larger than that of the (006) reflection (ca. 0.13 Å). In addition, it seems that there is a small peak splitting at 130 and 140 °C. This may support the assumption that an intermediate chemical transition occurs before thermal decomposition.

**Figure 5:**
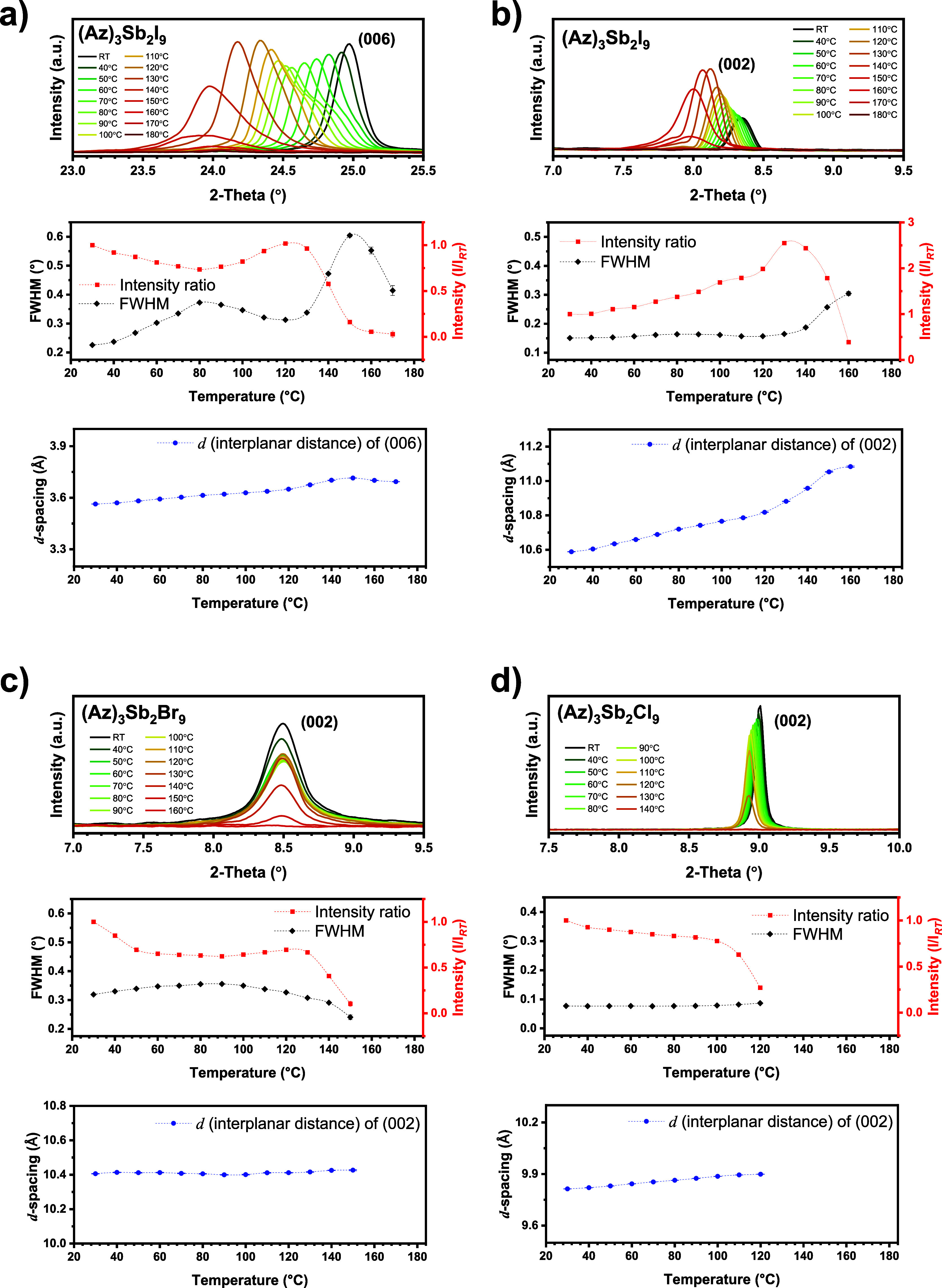
Temperature-dependent XRD patterns: (a) (006) reflection and (b) (002) reflection of (Az)_3_Sb_2_I_9_-DMSO, (c) (002) reflection of (Az)_3_Sb_2_Br_9_-DMSO, and (d) (002) reflection of (Az)_3_Sb_2_Cl_9_-DMF thin film on glass collected at different temperatures as well as the intensity of the reflections, their FWHM values, and the peak positions (2θ) as a function of temperature.

The (Az)_3_Sb_2_Br_9_-DMSO thin film shows thermal decomposition at 130 °C, which is lower than that of (Az)_3_Sb_2_I_9_ (Figure 5c). All XRD reflections vanish at 160 °C. The intensity of the (002) reflection slightly decreases with increasing temperature up to 50 °C. The reflection shows almost no intensity change between 50 and 130 °C, and the intensity declines above 140 °C. Interestingly, all reflections of this thin film do not show any shift in 2θ, which indicates that there is almost no thermal lattice expansion. This might be due to the very high thin-film densification, as seen in the SEM morphology in Figure 3a or due to clamping by the substrate.

The (002) reflection of the (Az)_3_Sb_2_Cl_9_-DMF film slightly shifts with temperature, namely, from 9.8134(2) to 9.8988(4) Å (Figure 5d). The decrease in the intensity ratio plot shows a significant change at 100 °C, which seems to correspond to the decomposition temperature point. All reflections disappear at 130 °C, which is a lower temperature than that in the (Az)_3_Sb_2_Br_9_-DMSO thin film. In the TG curves of the polycrystalline powders, (Az)_3_Sb_2_Cl_9_ demonstrates a degradation temperature point close to that of (Az)_3_Sb_2_Br_9_. We assume that the difference in decomposition temperature between polycrystalline powders and thin films is caused by the low thin-film density of (Az)_3_Sb_2_Cl_9_.

### Optical Properties

#### Polycrystalline Powders

Diffuse reflectance spectra of the (Az)_3_Sb_2_X_9_ polycrystalline powders were recorded by using UV–vis spectroscopy. Figure 6a–c shows Tauc plots for indirect and direct allowed transitions converted from the measured reflectance spectra via the Kubelka–Munk transformation, [*F*(*R*)*hv*]^*n*^ (*n* = 1/2 is assigned to an allowed indirect transition, and *n* = 2 is assigned to an allowed direct transition).^63^ We use extrapolation to roughly evaluate the band edges in the Tauc plots.^64^ For a direct transition analysis, (Az)_3_Sb_2_I_9_ displays a band edge at 2.26 eV in the band plot and at 2.11 eV for an indirect transition. It is assessed that there is a weak excitonic feature at 2.80 eV and a sub-band at 3.36 eV in both direct and indirect transition band plots. (Az)_3_Sb_2_Br_9_ displays a band edge at 2.82 eV in the direct transition band plot and at 2.63 eV in the indirect transition band plot. At 3.67 eV, it is estimated to have a sequential subgap in the direct transition band plot, but it is not obvious in the indirect transition band plot. (Az)_3_Sb_2_Cl_9_ shows a band edge at 3.50 eV in the direct transition band plot and at 3.26 eV in the indirect transition band plot.

**Figure 6:**
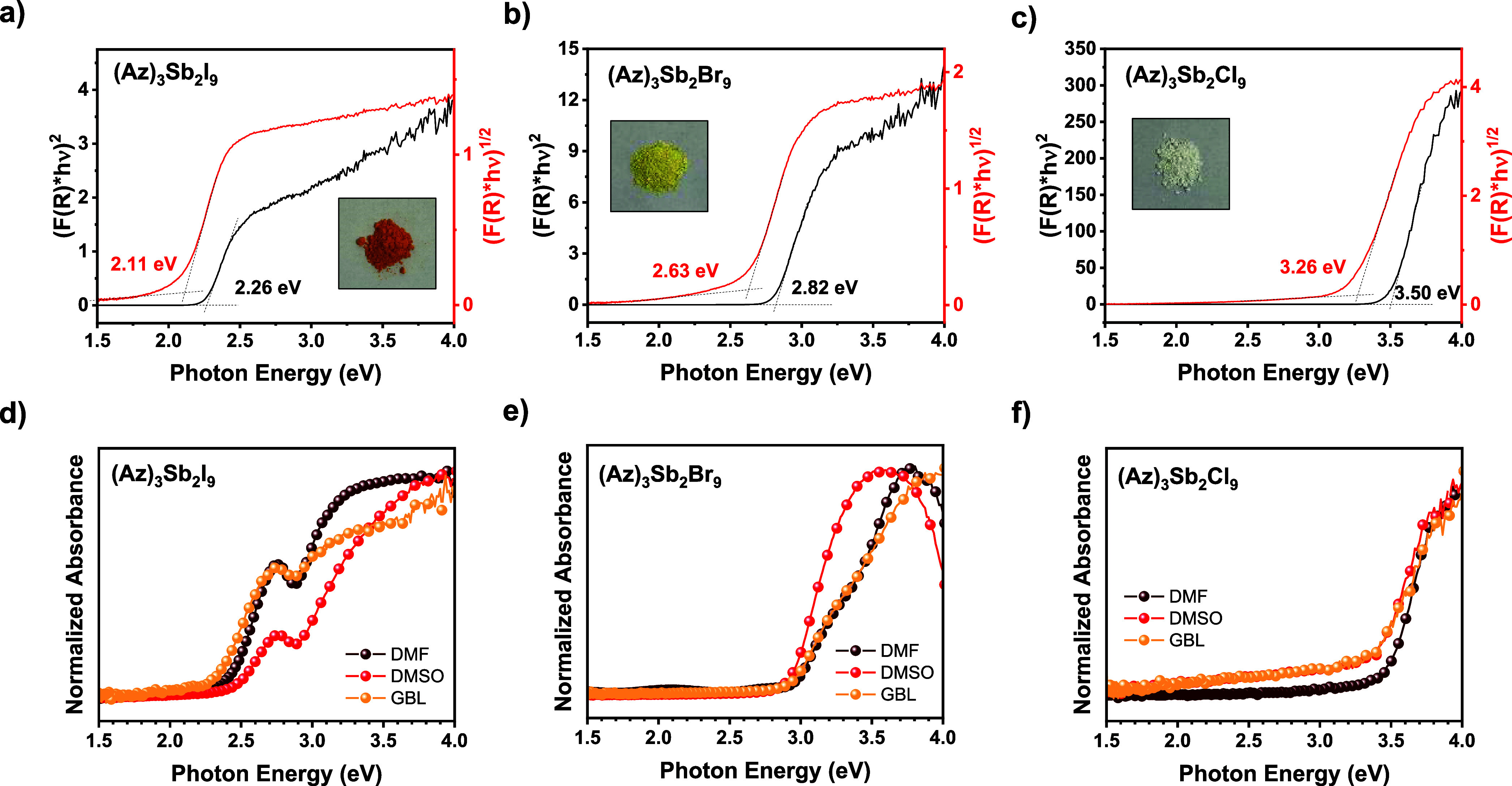
(a) Tauc plots converted by [*F*(*R*)*hv*]^*n*^ (direct allowed transition: *n* = 2, indirect allowed transition: *n* = 1/2) of the diffuse reflectance spectra of the polycrystalline powders of (a) (Az)_3_Sb_2_I_9_, (b) (Az)_3_Sb_2_Br_9_, and (c) (Az)_3_Sb_2_Cl_9_ via Kubelka–Munk transformation, absorption spectra of (d) (Az)_3_Sb_2_I_9_, (e) (Az)_3_Sb_2_Br_9_, and (f) (Az)_3_Sb_2_Cl_9_ thin films on cleaned glass substrates depending on the solvent used in the precursors (DMF, DMSO, and GBL).

#### Thin Films

Absorption spectra of (Az)_3_Sb_2_X_9_ thin films were measured by using UV–vis spectroscopy (Figure 6d–f). Further details are described in each paragraph for (Az)_3_Sb_2_X_9_ thin films depending on the solvent used (DMF/DMSO/GBL).

##### (Az)_3_Sb_2_I_9_

The spectrum results of the (Az)_3_Sb_2_I_9_ thin films show a deviation of the band edge compared with that of the Tauc plot of the polycrystalline powder. In Figure 6d, the different band edges are remarked: the (Az)_3_Sb_2_I_9_-DMF has the onset at 2.42 eV, (Az)_3_Sb_2_I_9_-DMSO at 2.45 eV, and (Az)_3_Sb_2_I_9_-GBL at 2.34 eV. The (Az)_3_Sb_2_I_9_-GBL has the closest band edge to that of the polycrystalline powder. An excitonic peak in all of the (Az)_3_Sb_2_I_9_ films is invariant at 2.75 eV. A sequential subgap, which might be an actual conduction band minimum, appears at 2.88 eV for all (Az)_3_Sb_2_I_9_ films. Only (Az)_3_Sb_2_I_9_-GBL has a second subgap at 3.67 eV. The excitonic peak of (Az)_3_Sb_2_I_9_ is relatively weak compared to the ones of (Az)_3_Bi_2_I_9_ and (Az)_3_Bi_2_Br_9_, which are 0D analogues reported in our previous paper.^54^ Due to the nonmetallic property of Sb, it is considered that Sb-based hybrid crystals usually induce excitonic dissipation as the form of nonradiative recombination under the organic-inductive effect.^65^ The weak excitonic band of (Az)_3_Sb_2_I_9_ is estimated to be featured by a dominant organic-inductive effect influenced by the relatively large dipole of Az^+^. The excitonic binding energy of the (Az)_3_Sb_2_I_9_ film is approximately 124 meV. In our previous report, the analogues (Az)_3_Bi_2_I_9_ and (Az)_3_Bi_2_Br_9_ exhibited binding energies of 225 and 400 meV, respectively.^54^ The 0D Cs_3_Sb_2_I_9_ is known to have an excitonic binding energy of about 175 meV, which is higher than (Az)_3_Sb_2_I_9_.^29^ Other related compounds, such as (FA)_3_Bi_2_I_9_ and (MA)_3_Bi_2_I_9_, have experimentally determined exciton binding energies of 260 and 320 meV, respectively.^66^

##### (Az)_3_Sb_2_Br_9_

The absorption onset of (Az)_3_Sb_2_Br_9_-DMSO occurs at 2.92 eV (Figure 6e). The (Az)_3_Sb_2_Br_9_-DMF and (Az)_3_Sb_2_Br_9_-GBL films have different band edges at 2.96 eV. (Az)_3_Sb_2_Br_9_-DMF shows a very weak broad band below the lower photon energy region than at the band edge. This broad band is not observed in the absorption spectra of (Az)_3_Sb_2_Br_9_-DMSO and (Az)_3_Sb_2_Br_9_-GBL. (Az)_3_Sb_2_Br_9_-DMSO does not show a specific excitonic feature in the absorption spectrum. The decline of the absorption at 3.75 eV is evident, and there is no absorption increasing point at the photon energy region higher than 3.75 eV. It might be thus plausible to explain that a broad excitonic band exists with a peak at 3.56 eV. (Az)_3_Sb_2_Br_9_-DMF and (Az)_3_Sb_2_Br_9_-GBL have a weak sub-band transition at 3.45 eV, with seemingly an excitonic peak at 3.29 eV. This peak of (Az)_3_Sb_2_Br_9_-GBL is weaker than that of (Az)_3_Sb_2_Br_9_-DMF. Based on the experimental result that (Az)_3_Sb_2_Br_9_-DMF has a poor crystallinity, it might be reasonable that a sub-band transition is caused by retaining impurities or secondary phases residues. At this point, we cannot identify, whether it is an excitonic peak or a band edge created by impurities.

##### (Az)_3_Sb_2_Cl_9_

In the absorption spectra of the (Az)_3_Sb_2_Cl_9_ films, the absorption onset of (Az)_3_Sb_2_Cl_9_-DMF is at 3.49 eV (Figure 6f). The (Az)_3_Sb_2_Cl_9_-DMSO and (Az)_3_Sb_2_Cl_9_-GBL have a slightly lower band edge at 3.44 eV. Every film has a subgap at 3.77 eV. The (Az)_3_Sb_2_Cl_9_-DMF almost coincides with the band edge of the Tauc plot of (Az)_3_Sb_2_Cl_9_ polycrystalline powder in the direct allowed transition. An excitonic peak only vaguely exists in both the Tauc plot of the (Az)_3_Sb_2_Cl_9_ powder and the absorption spectrum of (Az)_3_Sb_2_Cl_9_-DMF. If the excitonic state should necessarily emerge, the band transition at 3.81 eV in the absorption spectra of the thin films is presumed to be an excitonic band state.

### Urbach Energy Analysis

We analyzed the Urbach energy using powder diffuse reflectance spectra (transformed via the Kubelka-Munk function) and thin-film absorbance data (Figures S7 and S8). Urbach energy reflects the extent of disorder, such as amorphous regions or defects, manifested as the Urbach tail and broadening of the absorption edge.^67–70^ Higher values indicate greater disorder. The obtained Urbach energies for powders and thin films are summarized in Table S3. The results show that (Az)_3_Sb_2_I_9_ powder has lower disorder compared to its thin film, while for (Az)_3_Sb_2_Br_9_ and (Az)_3_Sb_2_Cl_9_, the thin films exhibit higher disorder than the powders. This is likely due to the excitonic binding energy in the (Az)_3_Sb_2_I_9_ thin films. We note that the fitted values for thin films may be less accurate due to the challenges in precisely defining the absorption coefficient. Nevertheless, the trend suggests that the solvent choice during precursor preparation influences film disorder: (Az)_3_Sb_2_I_9_-DMSO shows a Urbach energy similar to that of (Az)_3_Sb_2_I_9_-DMF, which is inconsistent with the higher crystallinity observed in XRD. SEM images reveal that (Az)_3_Sb_2_I_9_-DMF has a homogeneous grain distribution, which indicates that grains possibly adopt different orientations compared to (Az)_3_Sb_2_I_9_-DMSO. All (Az)_3_Sb_2_Br_9_ thin films display lower Urbach energies than their powders, which is expected due to the generally higher defect density in powders. Meanwhile, (Az)_3_Sb_2_Cl_9_-DMF has a low Urbach energy, while (Az)_3_Sb_2_Cl_9_-DMSO and (Az)_3_Sb_2_Cl_9_-GBL show higher values than that of the powder, supporting our XRD and SEM results of poor crystallinity in these films.

While other state-of-the-art perovskite materials show Urbach energies in the range of high crystalline systems like silicon or galium-arsenide, the Urbach energies reported here are 1 order of magnitude larger.^68–70^ The lower Urbach energies in the deposited thin films depending on the used solvent compared to the unordered powders underline the potential of the Az-compounds in thin-film applications with further improved deposition routines.

## Conclusions

It is demonstrated that the synthesis of (Az)_3_Sb_2_X_9_ polycrystalline powders and the deposition of thin films are feasible (summarized in Table 1). Az^+^ is well incorporated into the cationic site of the A_3_Sb_2_X_9_ lattice. The Az^+^ cation is randomly oriented at room temperature. Due to these dynamics of the molecular dipole, it may contribute to charge screening within this crystal. The (Az)_3_Sb_2_X_9_ forms a low-dimensional system: (Az)_3_Sb_2_I_9_ forms 0D dimers of the [SbI_6_] octahedra, and (Az)_3_Sb_2_Br_9_ and (Az)_3_Sb_2_Cl_9_ form a 2D corrugated layer structure at room temperature. Compared to those of (Az)_3_Bi_2_X_9_, which we reported previously, the crystal structures of (Az)_3_Sb_2_X_9_ are nearly identical to those of (Az)_3_Bi_2_X_9_, despite the difference in ionic radii: Sb^3+^ (0.76 Å) versus Bi^3+^ (1.03 Å).^54^ The (Az)_3_Sb_2_X_9_ thin films show high texture on cleaned glass substrates, which implies applicability to layered electronic devices. Thus, further structural study will be necessary with temperature dependence to identify potential Az^+^ dynamics. The optical band gaps of (Az)_3_Sb_2_X_9_ are slightly larger than those of (Az)_3_Bi_2_X_9_; however, their excitonic features are weaker (see Figure 7).^54^ The demonstrated feasibility of synthesis indicates that halide engineering in Az^+^ compounds could be an effective approach to achieving an optimal low band gap for photovoltaic applications. Accordingly, we propose the following strategic roadmap to realize and enhance device performance:^25,71,72^

(i) Exploring the feasibility of lowering the band gap through compositional engineering: systematically vary halide compositions and B-site divalent cations to tune the band gap energy. Use computational modeling and experimental synthesis to identify promising compositions that achieve optimal band gap values for the desired applications, such as in photovoltaics.
(ii) Assessing the photoactive properties through comprehensive characterization: perform impedance spectroscopy and Kelvin probe force microscopy to analyze charge transport, recombination rates, and defect states within the material. Measure photocurrent responses under simulated sunlight to evaluate the efficiency of charge generation and collection. Conduct transient photoluminescence and time-resolved spectroscopy to understand charge carrier dynamics and lifetimes.
(iii) Investigating band alignment and energy-level matching: carry out photoemission spectroscopy techniques, including X-ray photoelectron spectroscopy (XPS), ultraviolet photoelectron spectroscopy (UPS), and inverse photoemission spectroscopy (IPES), to determine the valence band maximum, conduction band minimum, and work function. Use these data to optimize the alignment of energy levels with other device components, such as the electron and hole transport layers.
(iv) Comparison with analogues and evaluating the suitability of the azetidinium molecular cation: systematically compare the structural, optical, and electronic properties of azetidinium-based perovskite/perovskite-related absorbers with those of analogues using other organic or inorganic A-site cations. Analyze how the molecular structure of azetidinium influences stability, defect tolerance, and charge transport, to assess its potential as a functional cation in device architectures.

**Figure 7:**
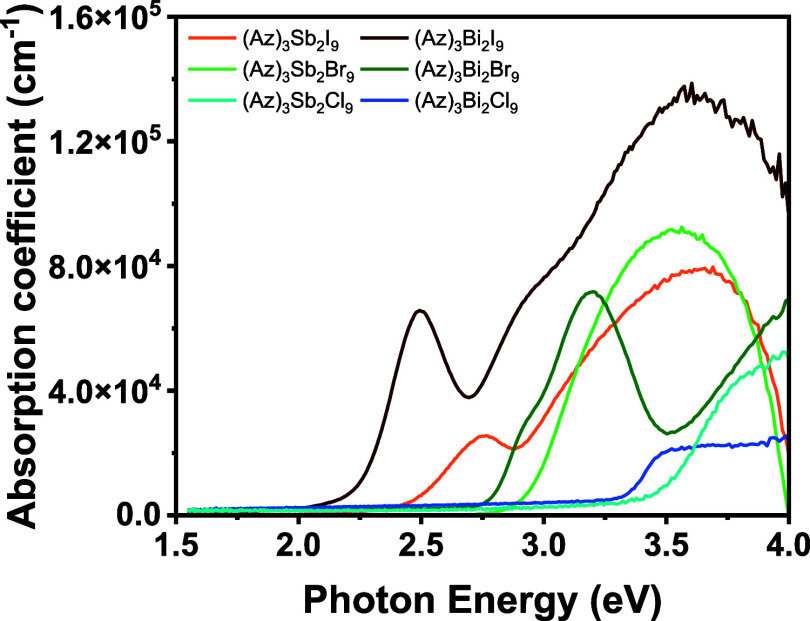
Absorption coefficient of (Az)_3_Bi_2_X_9_ and (Az)_3_Sb_2_X_9_ thin films.

## Supplementary Material



## Data Availability

Crystallographic data for (Az)_3_Sb_2_I_9_ have been deposited at the Cambridge Crystallographic Data Centre (CCDC) crystallographic database under the assigned number 2428821 (for both *Cmcm*/*Cmc*2_1_ symmetry). Crystallographic data for (Az)_3_Sb_2_Br_9_ have been deposited at the CCDC crystallographic database under the assigned number 2428823. Crystallographic data for (Az)_3_Sb_2_Cl_9_ have been deposited at the CCDC crystallographic database under the assigned number 2480297. No primary research results, software, or code have been included.

## Notes

Rietveld refinement of the crystal structure of the powder samples based on synchrotron PXRD data, crystallographic parameters, characterization information, electron density diagram of (Az)_3_Sb_2_Cl_9_, FT-IR spectrum, thin-film XRD patterns corresponding to the solvents used, UV-irradiation stability test results of the thin film, and Urbach energy diagram (PDF)
