# Supplementary material for: Fundamental Properties of Low-Dimensional Perovskite-Related Light Absorbers: [(CH2)3NH2]3Sb2X9 (X = I, Br, Cl), Azetidinium Antimony Halides
Source: ACS Omega. 2025 Dec 24;11(1):1101–13. doi: 10.1021/acsomega.5c08306 (PMC12809596; doi:10.1021/acsomega.5c08306)
Supplement: Supplementary file 1 [file ao5c08306_si_001.pdf]

Supporting Information

Fundamental properties of low-dimensional perovskite-related light absorbers: $[(CH_2)_3NH_2]_3Sb_2X_9$ (X=I, Br, Cl), azetidinium antimony halides

Young Un Jin,[a] Bernd Marler,[b] Andrei N. Salak,[c] Marianela Escobar Castillo,[a] Lars Leander Schaberg,[d] Erik Elkaïm,[e] Niels Benson,[d] and Doru C. Lupascu[a]*

a. Institute for Materials Science and Center for Nanointegration Duisburg-Essen (CENIDE), University of Duisburg-Essen, 45141 Essen, Germany

b. Institute of Geology, Mineralogy and Geophysics, Ruhr-University Bochum, 44780 Bochum, Germany

c. Department of Materials and Ceramics Engineering, CICECO-Aveiro Institute of Materials, University of Aveiro, 3810-193 Aveiro, Portugal

d. Institute of Technology for Nanostructures (NST), University of Duisburg-Essen, 47057 Duisburg, Germany

e. Synchrotron Soleil, L'Orme des Merisiers, Saint-Aubin, BP 48, Gif-sur-Yvette Cedex, 91192 France

*E-mail: young.jin@uni-due.de

Tel: +49-201-183-3051

Rietveld Refinement

The initial structure models were refined using the FullProf 2K program.[1] Hydrogen atoms of the azetidinium cations could not be located. Therefore, the occupancy factors of the carbon atoms used to describe the azetidinium cations were fixed at a value that covers also the scattering power of the hydrogen atoms and the additional electron of the nitrogen atom. For the refinement, soft distance restraints were used on the $[SbX_6]$ octahedra with d(Sb-Cl) = 2.80(3) Å, d(Cl-Cl) = 3.90(5) Å, d(Sb-Br) = 2.90(3) Å, d(Br-Br) = 4.00(5) Å, d(Sb-I) = 3.10(3) Å, d(I-I) = 4.30(5) Å and also appropriate soft distance restraints on carbon-carbon distances - depending on the symmetry of the particular structure - to refine the azetidinium cation as a pseudo rigid body. Six additional parameters were necessary to describe the anisotropic peak widths. The displacement parameters of the heavy atoms, antimony and the halides, were refined anisotropically.

[1] Rodríguez-Carvajal, J. Recent advances in magnetic structure determination by neutron powder diffraction. *Physica B: Condensed Matter* **1993**, *192*, 55–69.

SEM

Scanning electron microscopy (SEM) images were obtained from a Jeol (JSM-7500F) microscope. Every sample was Au-coated using a sputter coater.

FT-IR spectroscopy

Fourier transform infrared spectroscopy (FT-IR) for every polycrystalline powder was performed on an ALPHA Platinum FT-IR spectrometer (Bruker) in ATR (attenuated total reflectance) mode.

TG analysis

TG measurements for every polycrystalline powder were performed on a STA 449 F3 Jupiter from NETZSCH.

DSC measurement

DSC measurements for every polycrystalline powder were performed on a DSC 300 Caliris Classic from NETZSCH.

UV-Vis spectroscopy

Diffuse reflectance spectra of the polycrystalline powders and absorbance spectra of the thin films were measured on a Shimadzu UV2600 UV-vis spectrophotometer. Barium sulfate ($BaSO_4$) powder was used as a reference material in differential reflectance measurements.

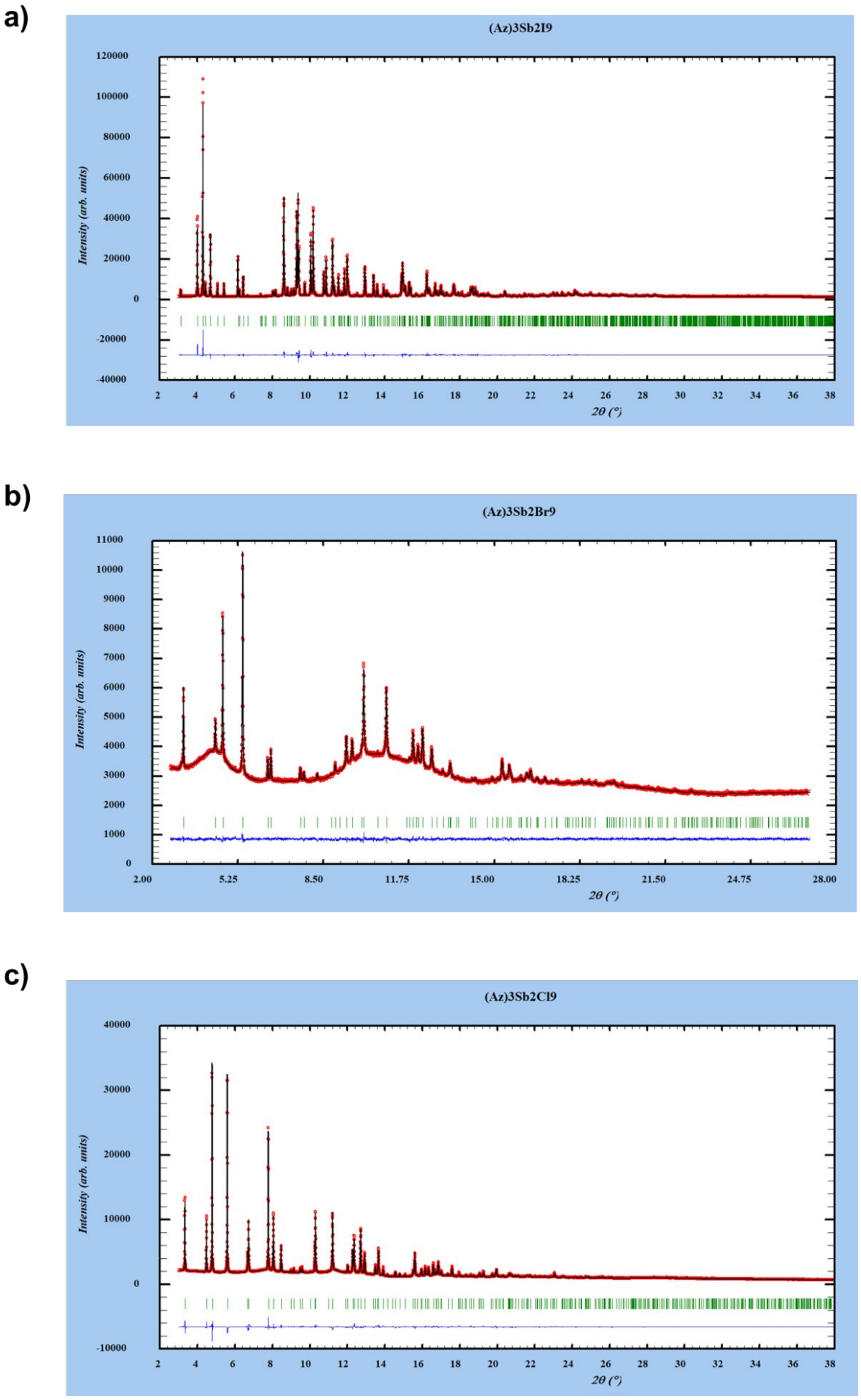

Figure S1. Final Rietveld plots of $(Az)_3Sb_2X_9$ structures, refined against Synchrotron X-ray diffraction data from polycrystalline powders of (a) $(Az)_3Sb_2I_9$ (b) $(Az)_3Sb_2Br_9$, and (c) $(Az)_3Sb_2Cl_9$.

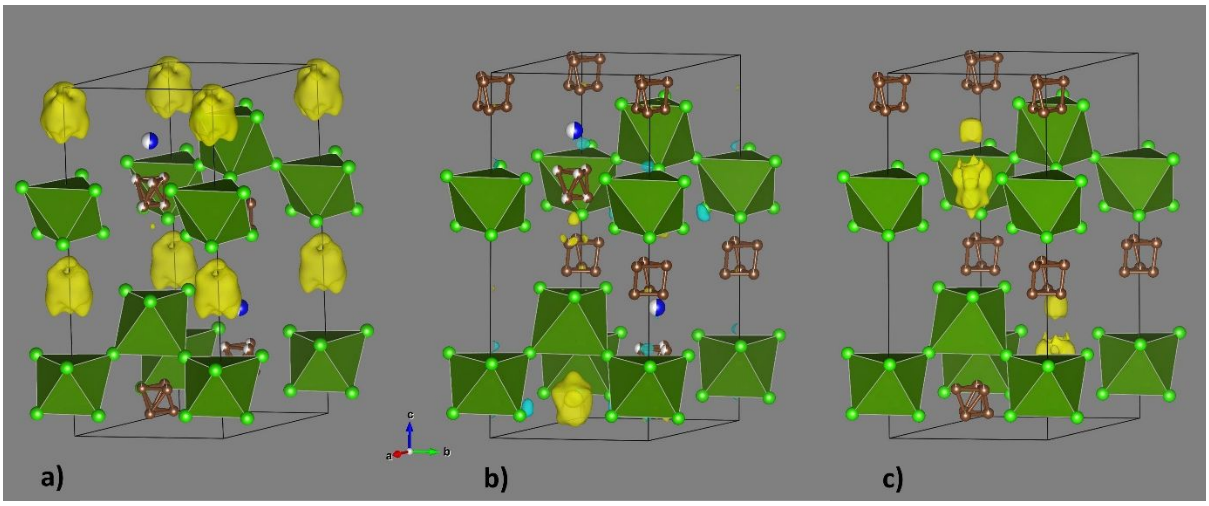

Figure S2. Remaining electron density (yellow clouds) calculated after removing the carbon atoms (shown as brown spheres) successively from the three azetidinium sites of the refined structure. The partly occupied site of the H_3O^+ cation is displayed in blue, a) removing the carbon atoms of Az1 from the structure model: F(min) = -0.47, F(max) = 0.89. Isosurface level set to 0.35 to display the electron clouds (the eight clouds are shared by 4 unit cells). b) removing the carbon atoms from Az2: F(min) = -0.46, F(max) = 1.00. Isosurface level set to 0.25 (a second cloud is hidden behind an octahedron). c) removing the carbon atoms from Az3: F(min) = -0.66, F(max) = 0.99. Isosurface level set to 0.41 (one of the two clouds is partly hidden behind an octahedron).

Table S1. Crystallographic parameters obtained from the structure refinements of $(Az)_3Sb_2X_9$.

Unit cell content	$(C_3H_8N)_{12}[Sb_8I_{36}]$	$(C_3H_8N)_6[Sb_4Br_{18}]$	$(C_3H_8N)_{5.2}(H_3O)_{0.8}[Sb_4Cl_{18}]$
Instrument	Beamline CRISTAL at SOLEIL Synchrotron		
Wavelength	0.58244 Å		
Sample holder	Glass capillary		
2Θ range of data used [°]	3.0 – 38.0	2.7 – 27.0	3.0 – 38.0
Step size [°2Θ]	0.003999	0.003999	0.003999
No. contributing reflections	4215	2671	2145
No. geometric restraints	31	49	41
No. structural parameters	46	38	40
No. profile parameters	14	14	13
FWHM at ca. 8°2Θ [°2Θ]	0.018-0.026	0.034-0.045	0.019 -0.031
R_I	3.25	4.08	3.86
R_{wp}	9.1	14.2	9.8
Space group	*Cmcm* (No. 63)	*P*31*c* (No. 159)	*P*31*c* (No. 159)
Crystal lattice	Orthorhombic	Trigonal	Trigonal
a [Å]	9.9737(1)	8.7467(1)	8.5763(1)
b [Å]	15.0178(1)	8.7467(1)	8.5763(1)
c [Å]	21.3485(2)	20.8753(3)	19.9627(3)
V_{UC} [Å^3]	3197.64(5)	1383.11(2)	1271.59(2)
Density (calc.) [g/cm^3]	3.290	2.787	1.944

Table S2. Comparison of the Rietveld refinement results of $(Az)_3Sb_2X_9$ according to the ambiguous symmetry

Material	$(Az)_3Sb_2I_9$		$(Az)_3Sb_2Br_9$		$(Az)_3Sb_2Cl_9$	
Space group symmetry	$C\,m\,c\,2_1$	C m c m	P 3 1 c	$P\,6_3\,m\,c$	P 3 1 c	$P\,6_3\,m\,c$
No. of structural variables	59	46	38	28	40	29
R(I)	3.26	3.25	4.08	5.64	3.86	5.84
R(wp)	9.32	9.07	14.2	14.6	9.79	12.9
Chi^2	21.3	20.2	2.07	2.20	6.22	10.83
Range of Sb-X distances [Å]	2.87 – 3.26	2.88 – 3.24	2.68 – 3.11	2.64 – 3.10	2.50 – 3.08	2.50 – 3.09
Range of X-X distances [Å]	4.11 – 4.41	4.16 – 4.40	3.78 – 4.40	3.73 – 4.44	3.53 – 4.35	3.41 – 4.41

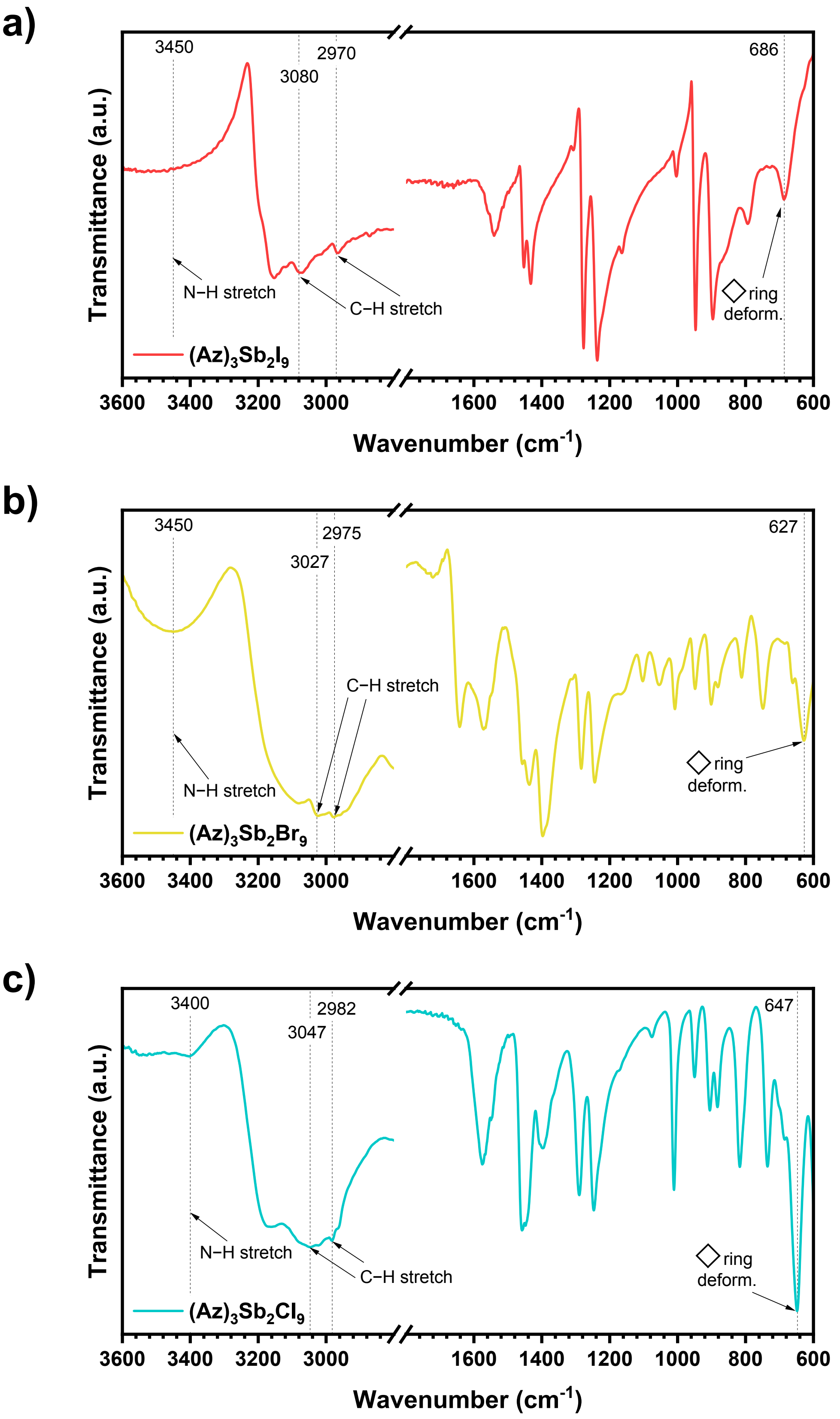

Figure S3. FT-IR transmittance spectra of (a) $(Az)_3Sb_2I_9$, (b) $(Az)_3Sb_2Br_9$, and (c) $(Az)_3Sb_2Cl_9$, and the line assignments of the azetidinium cation.

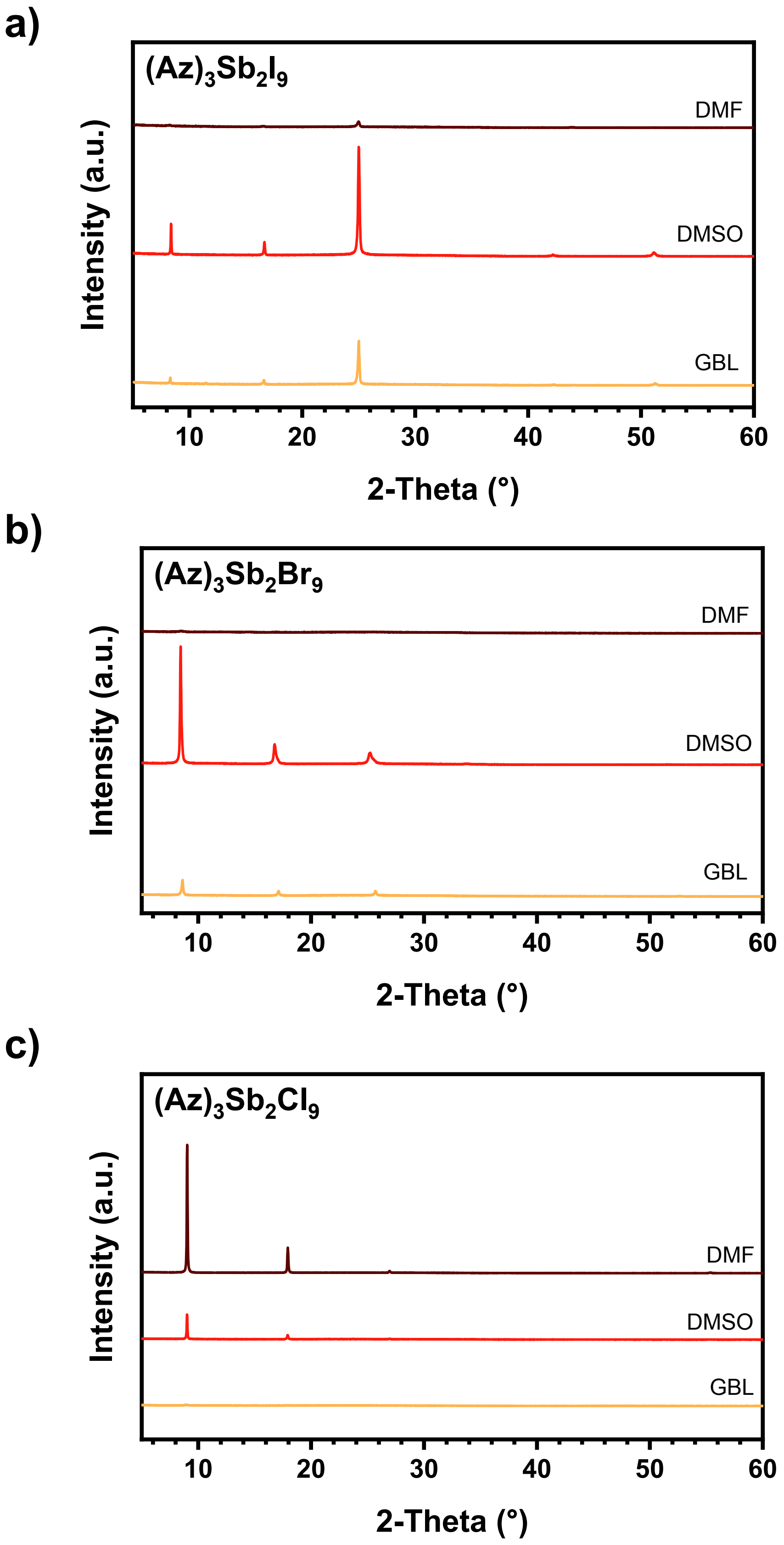

Figure S4. Thin film XRD patterns of the (a) $(Az)_3Sb_2I_9$, (b) $(Az)_3Sb_2Br_9$, and (c) $(Az)_3Sb_2Cl_9$ deposited on cleaned glass substrate depending on the solvent used in the precursors (DMF, DMSO, and GBL)

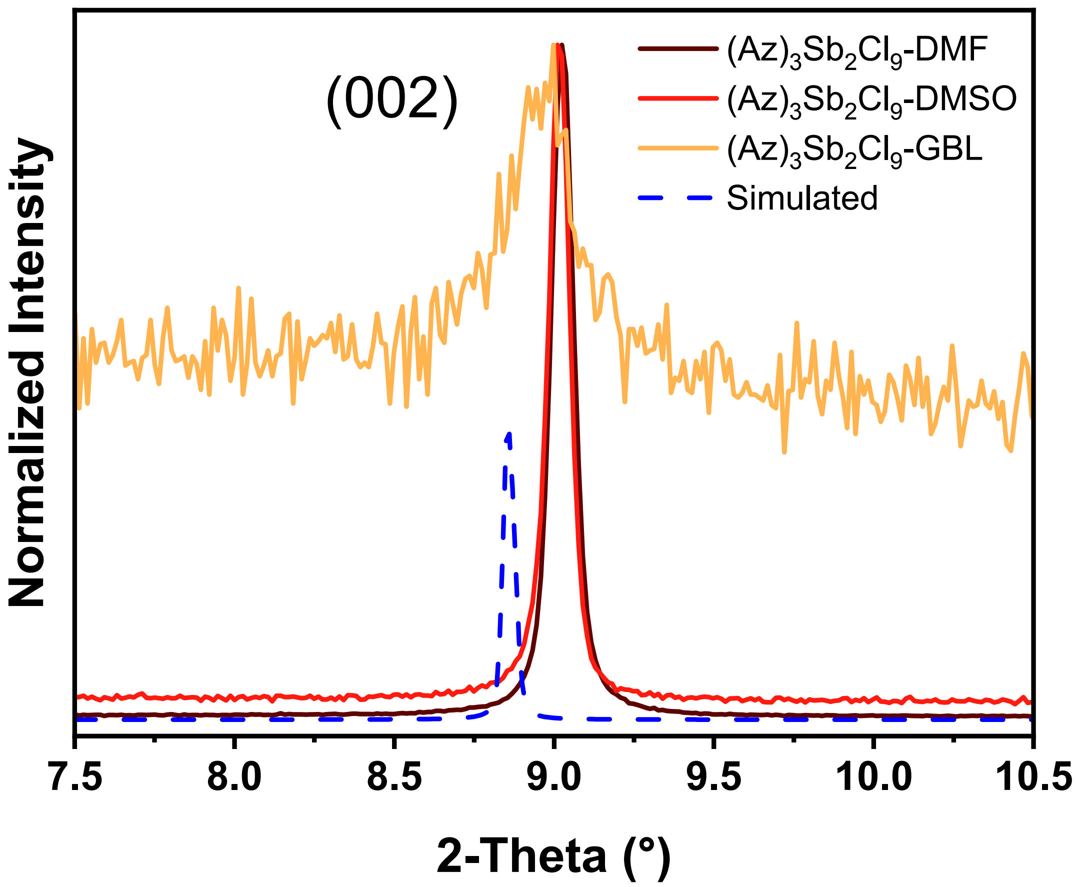

Figure S5. Comparison for the 2θ position of (002) reflection between $(Az)_3Sb_2Cl_9$-DMF, $(Az)_3Sb_2Cl_9$-DMSO, $(Az)_3Sb_2Cl_9$-GBL thin films and simulated XRD of $(Az)_3Sb_2Cl_9$ converted by Cu $K\alpha_{1/2}$ radiation.

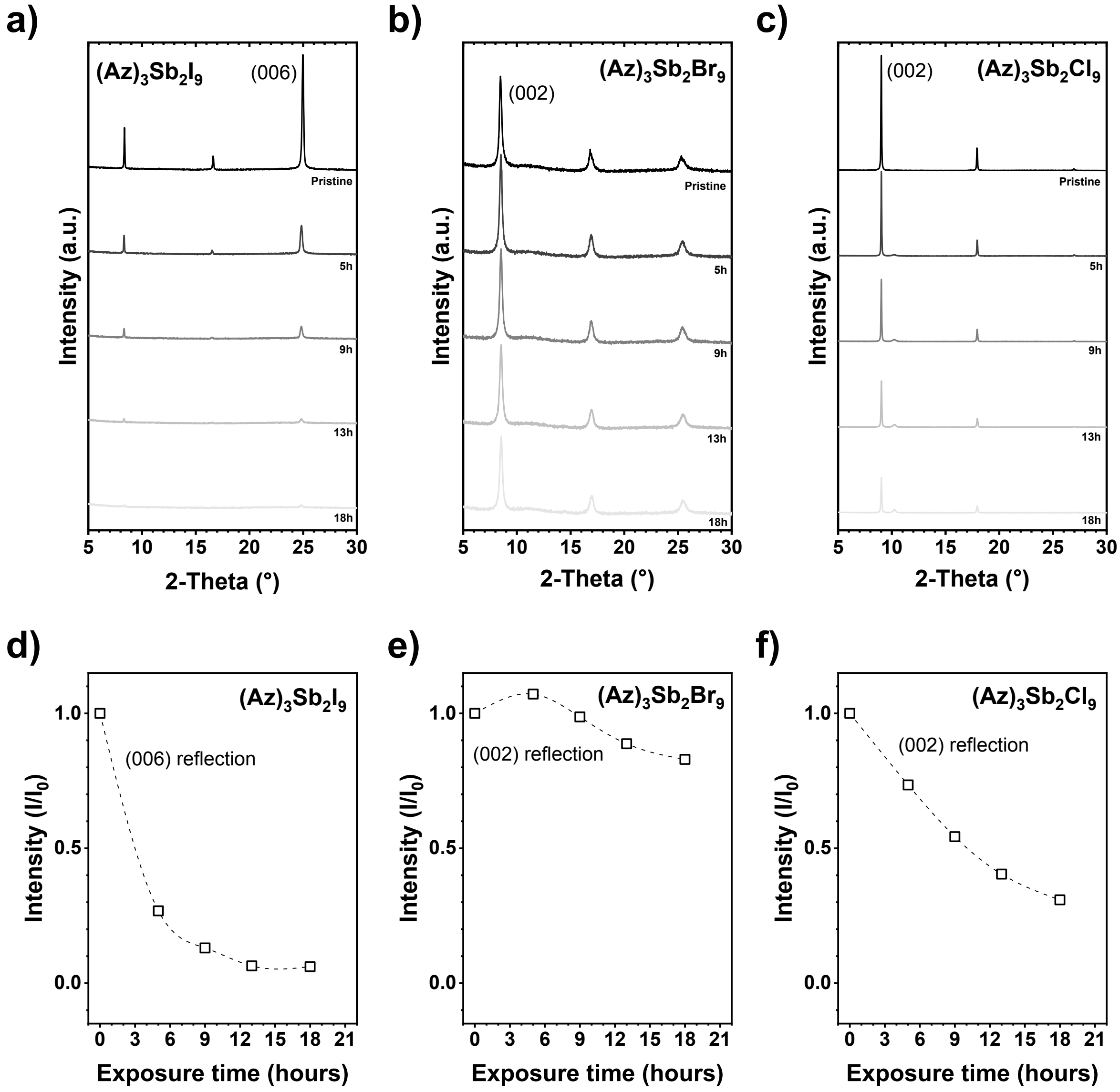

Figure S6. XRD patterns of the (a) $(Az)_3Sb_2I_9$-DMSO, (b) $(Az)_3Sb_2Br_9$-DMSO, and (c) $(Az)_3Sb_2Cl_9$-DMF thin films on cleaned glass substrate by UV-light exposure (0 h, 5 h, 9 h, 13 h, and 18 h), the intensity change diagram (I/I_0) of (d) the (006) reflection of the $(Az)_3Sb_2I_9$-DMSO, (e) the (002) reflection of the $(Az)_3Sb_2Br_9$-DMSO, and (f) the (002) reflection of the $(Az)_3Sb_2Cl_9$-DMF thin films. The diagrams exhibit an intensity decrease of $(Az)_3Sb_2I_9$-DMSO and $(Az)_3Sb_2Cl_9$-DMF, and a slight intensity decrease of $(Az)_3Sb_2Br_9$-DMSO by UV-light exposure time.

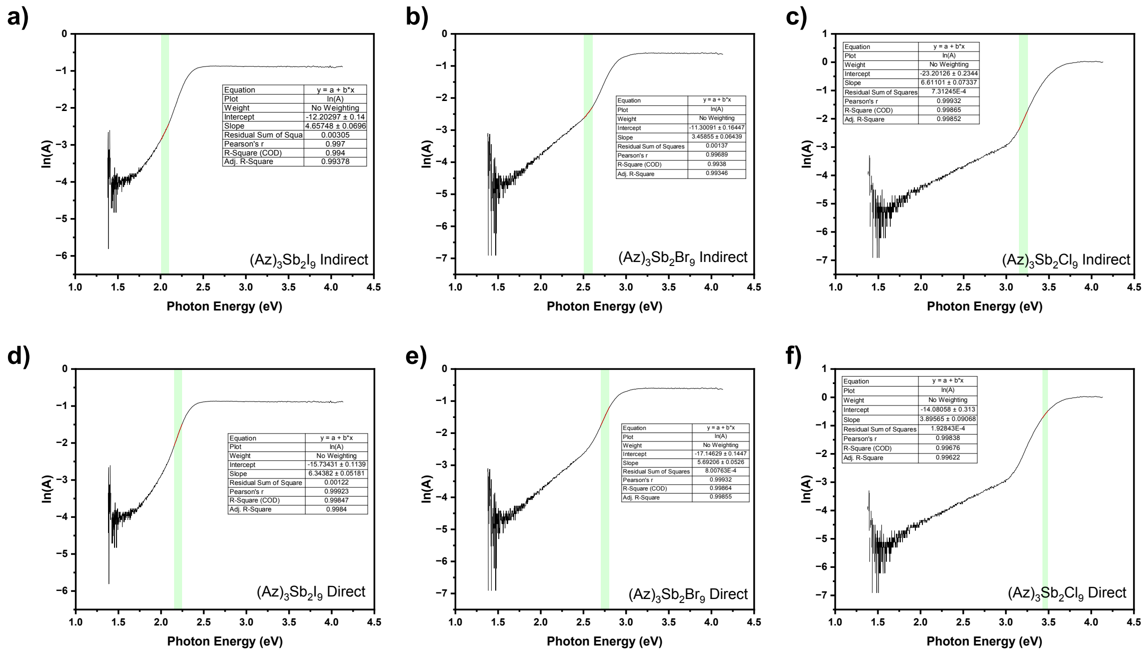

Figure S7. Urbach energy calculations from reflectance spectra transformed via the Kubelka-Munk function of the $(Az)_3Sb_2X_9$ powders: at (a-c) indirect allowed transition and (d-f) direct allowed transition.

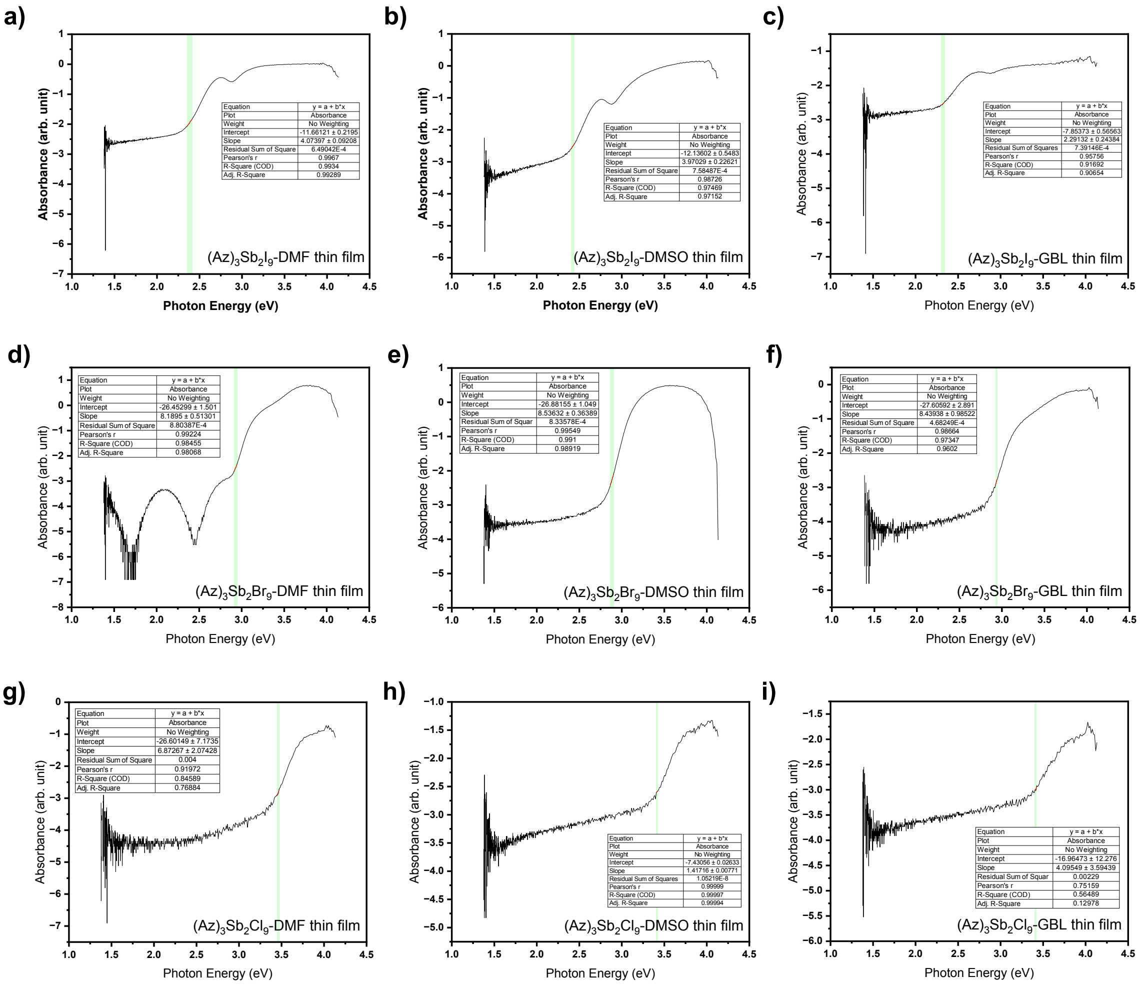

Figure S8. Urbach energy calculations from the absorbance of the thin films of (a-c) $(Az)_3Sb_2I_9$, (d-f) $(Az)_3Sb_2Br_9$, and (g-i) $(Az)_3Sb_2Cl_9$, depending on the used solvent: DMF, DMSO, and GBL.

Table S3. Urbach energy table calculated from the experimental optical spectra of $(Az)_3Sb_2X_9$.

Material	Sample type	Distinction	Bandgap energy (used for the fit)	Urbach energy
$(Az)_3Sb_2I_9$	Powder	direct	2.26	0.15763
		indirect	2.11	0.21471
$(Az)_3Sb_2Br_9$	Powder	direct	2.82	0.17568
		indirect	2.63	0.28914
$(Az)_3Sb_2Cl_9$	Powder	direct	3.5	0.2567
		indirect	3.26	0.15126
$(Az)_3Sb_2I_9$	Thin film	DMF	2.42	0.24546
		DMSO	2.45	0.25187
		GBL	2.34	0.43643
$(Az)_3Sb_2Br_9$	Thin film	DMF	2.96	0.12211
		DMSO	2.92	0.11715
		GBL	2.96	0.11849
$(Az)_3Sb_2Cl_9$	Thin film	DMF	3.49	0.1455
		DMSO	3.44	0.70564
		GBL	3.44	0.24417
